# Precision global health and comorbidity: a population-based study of 16 357 people in rural Uganda

**DOI:** 10.1098/rsif.2018.0248

**Published:** 2018-10-31

**Authors:** Goylette F. Chami, Narcis B. Kabatereine, Edridah M. Tukahebwa, David W. Dunne

**Affiliations:** 1 Department of Pathology, University of Cambridge, Tennis Court Road, Cambridge CB2 1QP, UK; 2 Schistosomiasis Control Initiative, Imperial College London, Norfolk Place, London W2 1PG, UK; 3 Bilharzia and Worm Control Programme, Vector Control Division, Ministry of Health, 15 Bombo Road, Kampala, Uganda

**Keywords:** networks, precision medicine, biosocial, symptoms, public health, global health

## Abstract

In low-income countries, complex comorbidities and weak health systems confound disease diagnosis and treatment. Yet, data-driven approaches have not been applied to develop better diagnostic strategies or to tailor treatment delivery for individuals within rural poor communities. We observed symptoms/diseases reported within three months by 16 357 individuals aged 1+ years in 17 villages of Mayuge District, Uganda. Symptoms were mapped to the Human Phenotype Ontology. Comorbidity networks were constructed. An edge between two symptoms/diseases was generated if the relative risk greater than 1, *ϕ* correlation greater than 0, and local false discovery rate less than 0.05. We studied how network structure and flagship symptom profiles varied against biosocial factors. 88.05% of individuals (14 402/16 357) reported at least one symptom/disease. Young children and individuals in worse-off households—low socioeconomic status, poor water, sanitation, and hygiene, and poor medical care—had dense network structures with the highest comorbidity burden and/or were conducive to the onset of new comorbidities from existing flagship symptoms, such as fever. Flagship symptom profiles for fever revealed self-misdiagnoses of fever as malaria and sexually transmitted infections as a potentially missed cause of fever in individuals of reproductive age. Network analysis may inform the development of new diagnostic and treatment strategies for flagship symptoms used to characterize syndromes/diseases of global concern.

## 1. Background

Biological and social factors determine individual variation in the onset and progression of disease pathologies [1–3]. Precision medicine seeks to incorporate such variation into the design of treatments for individuals [4,5]. The ultimate aim is to produce tailored clinical treatments for individuals or subsets of a population based on their genetic, environmental and lifestyle factors. Thus far, due to rapid advances in deoxyribonucleic acid sequencing, the availability of large genomic datasets, and electronic medical records (EMRs), the study of precision medicine has focused on genetic variation [6]. Genome-wide and phenome-wide studies investigate many, sometimes millions of genetic variants against phenotypic traits/outcomes and vice versa [6]. These data-driven methods reveal causal pathways of a disease and common genetic variants of seemingly unrelated phenotypic abnormalities [7].

Network medicine provides a systems-based approach for the study of precision medicine [8–10]. Pairwise relationships between a type of molecular marker or phenotype, such as genes [11], proteins [12], metabolites [13], symptoms [14] and clinical diagnoses [15], are graphed. The organizational properties of these irregular structures (complex networks), the correlation between different networks, and the responses of each network to perturbations are studied. A key structural feature of these biological networks is their high modularity; nodes tend to cluster together due to shared disease mechanisms and/or phenotypes [8,16]. Analysis of such clusters has informed patient stratification for randomized-controlled trials [17], identified unusual comorbidities [15], tracked disease progression [18], and revealed new disease mechanisms, biomarkers, and drug targets [11,13,19]. The study of genetic or other molecular variation is insufficient to enable the use of precision/network medicine in routine clinical practice. A better understanding of environmental and lifestyle predispositions to disease is needed to holistically capture individual variation in disease pathologies and to enable administration of tailored clinical treatments within routine medical practices [4]. A few studies [15,18,20,21] of clinical diagnoses have examined variation in network structure by age, gender and race. Networks of clinical diagnoses in Austria were shown to display the highest modularity (clustering into distinct anatomical disease categories) in adolescence and adulthood [21]. African Americans have been shown to experience a greater number [20] and different set [15] of comorbid diseases when compared to individuals of European descent.

Precision global health, albeit a nascent concept, extends ideas of precision medicine from individuals to communities [22–26]. The goal is to use biological, social and geographical information to tailor interventions to target communities in low-income countries. This data-driven approach to community health requires advanced computational and statistical tools that can inform practical, low-cost interventions [23]. Currently, satellite images are used (http://www.map.ox.ac.uk, accessed 9 October 2018) and epidemiological surveys from research datasets are assembled (http://www.thiswormyworld.org, accessed 9 October 2018) to map the location of parasite vectors for malaria and lymphatic filariasis, intermediate snail hosts for schistosomiasis, and climatic conditions required for soil-transmitted helminths. These spatial analyses improved not only estimations of the number of people at risk for these infections, but also the targeted distribution of bed nets, antimalarial drugs and preventive chemotherapies. With these advances in geotargeting, the next step towards precision global health is to move beyond cartography and to consider individual characteristics. An understanding of how health status varies by demographic, behavioural and social factors is essential to ensure the delivery of global health interventions to the right communities.

Analyses needed to inform precision global health in low-income countries also address current gaps in precision medicine [22,24]. To our knowledge, non-hypothesis based, data-driven methods have not been used to examine how socioeconomic inequalities and preventative health behaviours shape disease pathologies. These factors confound symptoms that are used by lay health workers to refer patients for formal diagnoses or by patients to seek medical care [1]. In rural poor settings, this problem is exacerbated by the presence of diverse comorbidities and weak health systems. Published studies [6,13,15,17,18,21] focus on individuals of European descent within hospitals of high-income countries where EMRs are available. Here we present the first study of comorbidity networks to inform the tracking of symptoms in low-income countries. The goal of this study is not to provide definitive diagnoses of diseases, but instead to investigate patient-identified symptoms that influence disease surveillance measures, treatment referrals, health-seeking behaviours and disease management campaigns. Consequently, we anticipate that this study may lead to improved diagnostic/treatment strategies in resource-poor settings.

## 2. Methods

### 2.1. Study design

We collected health, socioeconomic and behavioural data on nearly all (16357) individuals aged 1+ years in 17 rural villages of Mayuge District, Uganda. Some of the known infections widely affecting individuals in our study area include malaria, *Schistosoma mansoni* (intestinal schistosomiasis), hookworm and pneumonia [27,28]. Symptoms were the focus of this study because symptoms are the highest level of phenotype, i.e. most directly translatable to clinical practice and health-seeking behaviours of patients. In our rural poor study villages, self-reports are the only available health data; no EMRs exist. Moreover, many individuals in rural villages do not seek medical care from government health centres due to limited drug stocks, distance to clinics and costs of treatment [29]. Accordingly, the household head and/or wife were asked to report any illness experienced by all members of their household within the three months preceding the study survey. Lay surveyors were employed to record responses and, unlike conventional verbal autopsies [30], respondents were not prompted to elaborate on any particular symptoms that may be related to specific diseases. We used an unsupervised elicitation approach to collect health data in order to understand what comorbidity exists from the perspective of the study population. The self-reports predominantly provided lists of symptoms, but also self-diagnoses of diseases. For clarification, the term ‘diarrhoea’ here is henceforth used to refer to the reporting of the following symptom: 3+ watery stools within a day. Injuries also were reported since all medical conditions perceived as a health problem were recorded. All self-reports are provided in the electronic supplementary material, External Database S1.

### 2.2. Flagship symptoms

We defined a flagship symptom as a prevalent symptom within communities of interest that is used for disease surveillance, disease outbreak responses or routine presumptive treatment campaigns [31–33]. Here, we focus on reports of fever. There is a need to gain a better understanding of the self-reported symptoms that co-occur with fever. These complex, perceived febrile syndromes are understudied yet are of international importance. The recognition of fever by patients or lay health workers is currently one component, for example, of suspected case identification, self-monitoring, patients presenting to clinical facilities, and contact tracing during deadly Ebola or cholera outbreaks [31,32]. In addition to emergency/epidemic responses, self-reported fever is used in the World Health Organization's (WHO) community case management (CCM) strategies within the integrated management of childhood illness (ICMI) [33]. CCM in ICMI relies on clinically untrained (lay) community volunteers who move from household to household to provide antibiotics, antimalarials and oral rehydration salts for children aged less than 5 years. This administration is performed based on a caretaker's self-reports of their child's fever. Other routine uses of self-reported fever include the monitoring of severe adverse events during en masse treatment or vaccination campaigns [34]. Overall, self-reports of fever facilitate early detection and case management in areas with limited technological resources or clinical expertise.

### 2.3. Biosocial determinants

Four sub-group analyses were conducted of the full study population. Age and gender were investigated; demographics of the study population are provided in table 1. The type (quality) of healthcare accessed was recorded and included private clinics (poor), government centres (high), and both private and government care (moderate). Hence, moderate quality care includes multiple types of healthcare accessed. A more detailed description of the types of healthcare is provided in the electronic supplementary material. In brief, private clinic care includes informal/traditional healers as well as private drug shops and private clinics. Government healthcare includes only formal administrative medical units in Uganda. Government healthcare is considered of a higher clinical standard than private care due to the more frequent use of formal diagnostics before treatment administration, the presence of clinically qualified personnel, and government monitoring/regulation [35]. The first component from polychoric and tetrachoric principal component analyses (PCA) [36,37] was used, respectively, to reduce the dimensionality of many indicators of socioeconomic status (SES) and water, sanitation, and hygiene (WASH) behaviours. PCA scores were calculated at the household level and ranked from low (poor), middle (moderate) and high (cut-offs 33.33 and 66.67 percentiles) to construct SES and WASH indices.

**Table 1.** Study population by age and gender. This table presents the breakdown of the study population, including all individuals surveyed (16 357). Though respondents were only asked to name individuals in their household aged 1+, there were 70 people aged less than 1 year reported. These 70 people (2.85%; 70/2452) were included in the age category of 1–4 years.

age (years)	gender		total	% of all
	female	male		
1–4	1228	1224	2452	14.99%
5–14	2906	2851	5757	35.20%
15–49	3572	3400	6972	42.62%
50+	552	624	1176	7.19%
total	8258	8099	16 357	100%

Eleven SES variables were measured at the household level [38]. Home quality score was measured as the sum of scores for floor, wall and roof materials. Materials were ranked from 1 to 4 and summed. In order of rankings, the materials were as follows. Floor materials included mud, plastic, wood planks and bricks/cement. Wall materials were mud/sticks, plastic, metal and bricks/cement. Roof materials were grass/thatched, sticks, plastic and metal. Education was an ordinal variable measuring the highest level of education completed by any member of the household, including no (level 0), primary (levels 1–7), secondary (senior classes 1–6; levels 8–13) and higher education (diploma = level 14, some university = level 15, completed university = level 16). The local council is the village government, comprising nine positions: chairman, vice chairman, secretary, defence, gender secretary, disabled secretary, youth council, information secretary and elderly secretary. Membership to the council was measured as a binary variable from two sources: a household survey question asking if any member of the home is currently or was previously on the local council and a researcher-led survey interviewing the head of the village (chairman) and recording the names of all current council members. ‘Other status’ (binary) was defined as a household member currently or previously holding a position as a religious, tribe, or clan leader or as a member of the village health team. Religion was measured as a binary indicator. The minority religion in the study area was Muslim (1016/3491 households). The base category for religion was Christian (2300/3491 households) with very few respondents indicating another religion (108/3491 households). Two dummy indicators for occupation were constructed. Households were classified as fishing and/or farming households if anyone in the home received the majority of their income from that particular occupation; farms included rice paddies. These two classifications represent the most common jobs in the study area [27,38].

We defined WASH according to international guidelines, i.e. using the WHO and United Nation's Children's Fund's Joint Monitoring Programme definitions (www.wssinfo.org, accessed 9 October 2018). Seven binary WASH indicators were used. Questions on improved water and sanitation were presented as shown in international surveys, e.g. the World Health Survey (WHS) (http://www.who.int/healthinfo/survey/en/, accessed 9 October 2018) and Multiple Indicator Cluster Surveys (http://mics.unicef.org, accessed 9 October 2018). In our study area, improved water for drinking, cooking, or bathing consisted of access to and use of piped water, village taps, boreholes, protected springs, or rainwater collection into tanks. Another important aspect of safe water is quantity. In the same manner described in the WHS, household water quantity was recorded as positive if there were at least 20 litres of water per person (about one jerry can) available per day for drinking, cooking and personal hygiene. Improved sanitation in the study villages included ownership of a private, covered pit latrine at home that was not shared with any other households. Two indicators for hygiene were used. The frequency of hand washing with soap is the best proxy indicator of hygiene (www.wssinfo.org). However, because we neither observed hand washing—a difficult task—nor had data on available hand washing stations (an uncommon feature in our study villages), we asked households about the availability of soap in a manner unrelated to hand washing in attempt to elicit an unbiased response. Soap availability was coded as positive if a household washed their jerry cans with soap before storing water or used soap when washing clothes or bathing in Lake Victoria. A second indicator of hygiene concerned water treatment. This question was taken from the WHS. We defined water treatment as any chemical or physical action to make water safer to drink [39]. Water treatment was positive if a household used bleach/chlorine, boiled water, strained water through a cloth, or used a water filter.

A breakdown of all SES and WASH indicators by first component PCA indices is provided in tables 2 and 3. Additional method details are provided in the electronic supplementary material (figures S1–S8 and tables S1–S4).

**Table 2.** Summary of socioeconomic status (SES) variables by SES index categories.

	socioeconomic index category					
	low		middle		high	
	33.35% (1141/3421) households		33.29% (1139/3421) households		33.35% (1141/3421) households	
	25.38% (4068/16 026) individuals		32.33% (5182/16 026) individuals		42.48% (6776/16 026) individuals	
variable	per cent; *mean*	frequency; *s.d.*	per cent; *mean*	frequency; *s.d.*	per cent; *mean*	frequency; *s.d.*
council	0.44	5/1141	3.51	40/1139	24.63	281/1141
other status	0.18	2/1141	0.61	7/1139	6.13	70/1141
majority tribe	25.94	296/1141	34.33	391/1139	43.73	499/1141
Muslim	31.46	359/1141	29.06	331/1139	28.48	325/1141
fishing household	58.28	665/1141	21.95	250/1139	8.24	94/1141
farming household	18.58	212/1141	46.36	528/1139	69.50	793/1141
education	*5*.*97*	*2.78*	*6*.*79*	*2.85*	*7*.*95*	*2.98*
home ownership	62.14	709/1141	93.59	1066/1139	99.39	1134/1141
home electricity	1.67	19/1141	4.39	50/1139	11.31	129/1141
home quality score	*4*.*98*	*3.01*	*6*.*17*	*3.27*	*8*.*43*	*3.01*
years in village	*6*.*68*	*5.90*	*12*.*02*	*8*.*60*	*22*.*27*	*11.80*

**Table 3.** Summary of water, sanitation and hygiene (WASH) variables by WASH category.

	water, sanitation and hygiene (WASH) index category					
	poor		moderate		high	
	31.37% (1095/3491) households		29.50% (1030/3491) households		39.13% (1366/3491) households	
	32.46% (5309/16 357) individuals		30.48% (4986/16 357) individuals		37.06% (6062/16 357) individuals	
variable	per cent	frequency	per cent	frequency	per cent	frequency
improved drinking water	7.40	81/1095	94.27	971/1030	99.05	1353/1366
improved cooking water	0.09	1/1095	1.84	19/1030	70.42	962/1366
improved bathing water	0	0/1095	0.49	5/1030	49.71	679/1366
water availability/quantity	52.15	571/1095	54.56	562/1030	77.82	1063/1366
water treatment	59.18	648/1095	38.83	400/1030	21.60	295/1366
soap availability	40.55	444/1095	50.39	519/1030	12.59	172/1366
improved sanitation	7.49	82/1095	12.23	126/1030	16.91	231/1366

### 2.4. Network construction

**Figure 1.**
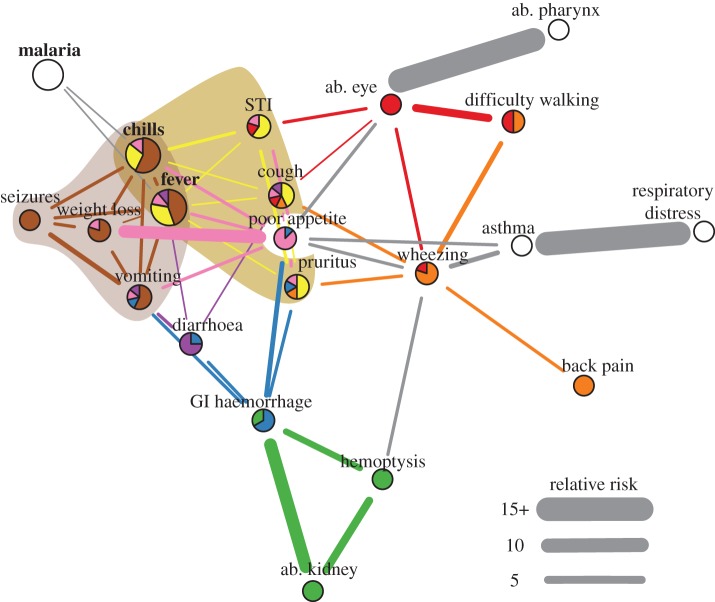
Full population comorbidity. Nodes represent reported symptoms/diseases; HPO terms are shown for symptoms. Abbreviations: ab. ‘abnormality of the’, GI ‘gastrointestinal’ and STI ‘sexually transmitted infection’. Edges shown had relative risk (RR) > 1, *ϕ* > 0 and *q*-value < 0.05. Widths of edges are capped at a RR of 15. Colours correspond to link clusters; grey edges and white nodes were not grouped into a link cluster. The main clusters for fever are shaded as a visual aid. A main cluster was where a majority of edges for fever were grouped. If there was no majority, all clusters with greater than 1 edge with fever were highlighted/shaded. Bolded and enlarged nodes were the symptoms/diseases that were most often co-reported with another symptom/disease. Among individuals who reported at least two symptoms/diseases (8718), malaria, fever and chills were mentioned by 70.19% (6119), 45.94% (4005) and 37.46% (3266) of people, respectively. A force-directed layout for comorbidity networks was used; the most connected symptoms/diseases are shown in the centre of the graphs.

**Figure 2.**
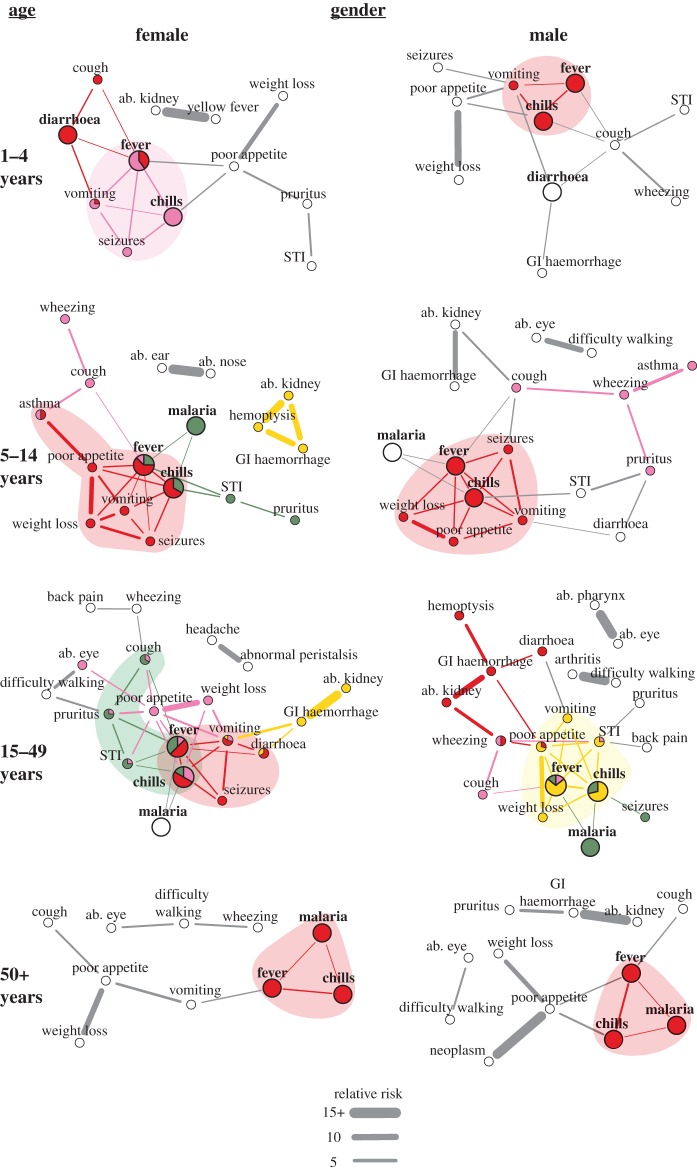
Comorbidity by age and gender. Nodes represent reported symptoms/diseases; HPO terms are shown for symptoms. ab. ‘abnormality of the’, GI ‘gastrointestinal’ and STI ‘sexually transmitted infection’. Edges shown had relative risk (RR) > 1, *ϕ* > 0 and *q*-value < 0.05. Widths of edges are capped at a RR of 15. Colours correspond to link clusters; grey edges and white nodes were not grouped into a link cluster. The main clusters for fever are shaded as a visual aid. A main cluster was where a majority of edges for fever were grouped. If there was no majority, all clusters with greater than 1 edge with fever were highlighted/shaded. Bolded and enlarged nodes were the symptoms/diseases that were most often co-reported with another symptom/disease. A force-directed layout for comorbidity networks was used; the most connected symptoms/diseases are shown in the centre of the graphs.

**Figure 3.**
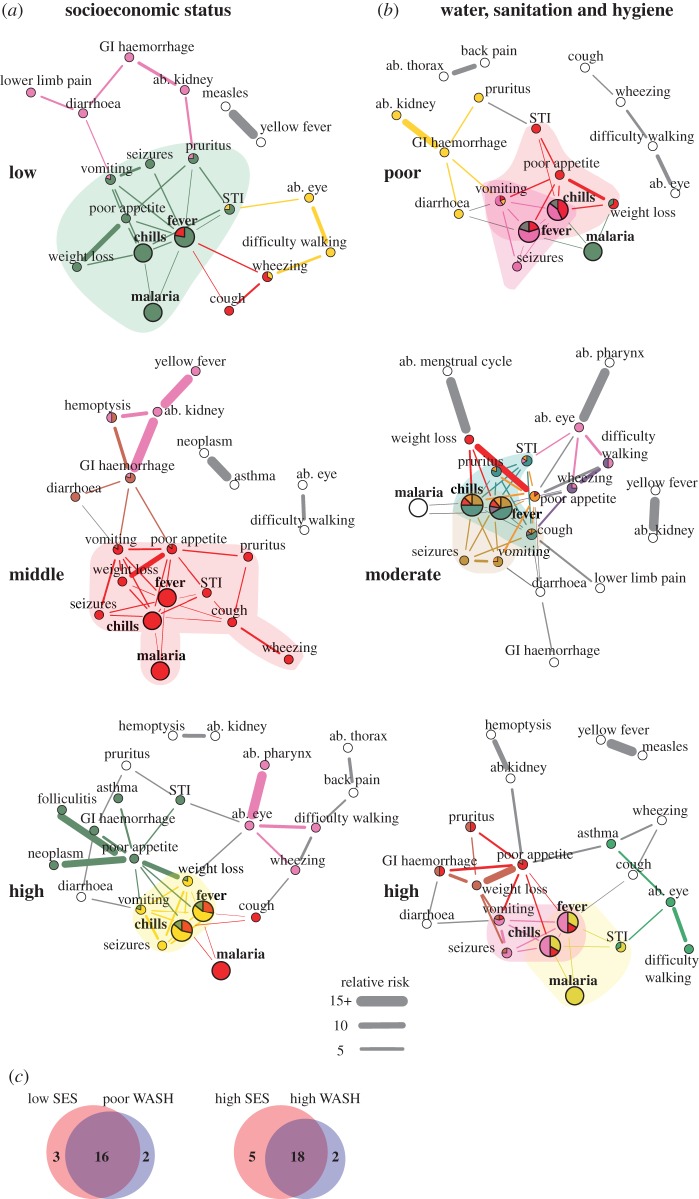
Comorbidity by SES and WASH. Nodes represent reported symptoms/diseases; HPO terms are shown for symptoms. ab. ‘abnormality of the’, GI ‘gastrointestinal’, STI ‘sexually transmitted infection’, SES ‘socioeconomic status’ and WASH ‘water, sanitation and hygiene’. Edges shown had relative risk (RR) > 1, *ϕ* > 0 and *q*-value < 0.05. Widths of edges are capped at a RR of 15. Colours correspond to link clusters; grey edges and white nodes were not grouped into a link cluster. The main clusters for fever are shaded as a visual aid. A main cluster was where a majority of edges for fever were grouped. If there was no majority, all clusters with greater than 1 edge with fever were highlighted/shaded. Bolded and enlarged nodes were the symptoms/diseases that were most often co-reported with another symptom/disease. A force-directed layout for comorbidity networks was used; the most connected symptoms/diseases are shown in the centre of the graphs. (*a*) Comorbidity by SES category. (*b*) Comorbidity by WASH category. (*c*) Overlap in symptoms/diseases (nodes) by SES and WASH categories.

**Figure 4.**
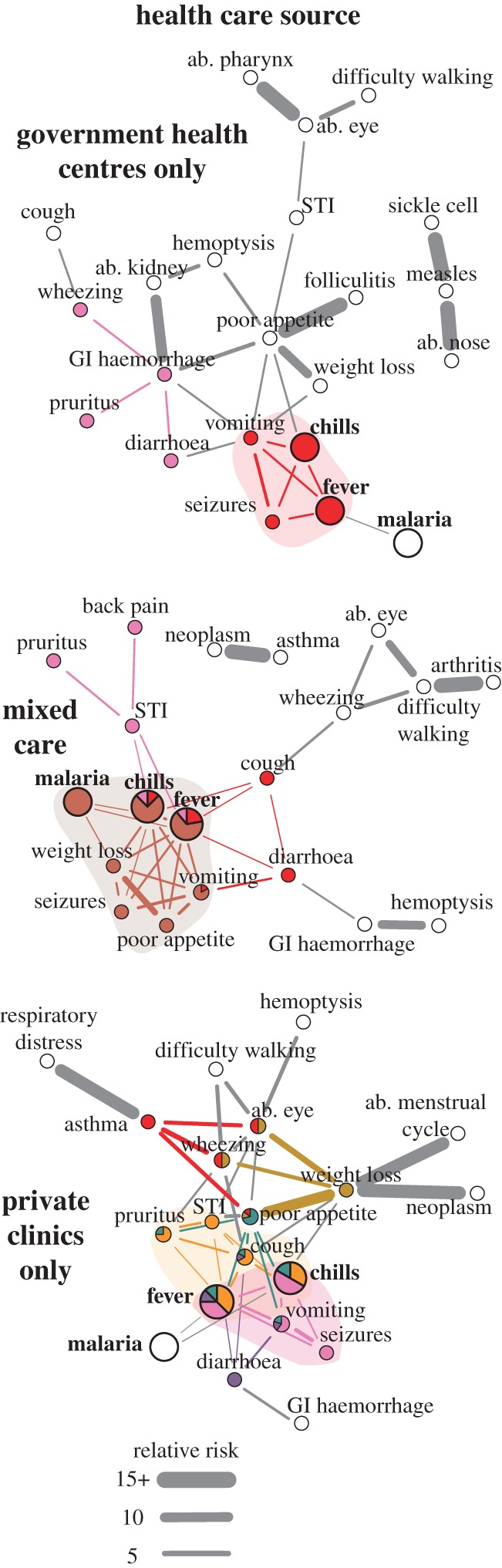
Comorbidity by type of healthcare accessed. Nodes represent reported symptoms/diseases; HPO terms are shown for symptoms. ab. ‘abnormality of the’, GI ‘gastrointestinal’ and STI ‘sexually transmitted infection’. Edges shown had relative risk (RR) > 1, *ϕ* > 0 and *q*-value < 0.05. Widths of edges are capped at a RR of 15. Colours correspond to link clusters; grey edges and white nodes were not grouped into a link cluster. The main clusters for fever are shaded as a visual aid. A main cluster was where a majority of edges for fever were grouped. If there was no majority, all clusters with greater than 1 edge with fever were highlighted/shaded. Bolded and enlarged nodes were the symptoms/diseases that were most often co-reported with another illness. A force-directed layout for comorbidity networks was used; the most connected symptoms/diseases are shown in the centre of the graphs.

To generate interoperable, computable terms, the laymen symptom reports were mapped to entries in the Human Phenotype Ontology (HPO) [40]. We constructed a directed acyclic graph to collapse matched terms and to ensure each HPO entry was distinct, i.e. no entry was a subclass of another term in our dataset. HPO mapping data are provided in the electronic supplementary material, External Database S1. We defined comorbidity as co-reported symptoms/diseases, though it should be noted a number of other definitions exist [41]. Comorbidity networks were constructed in Python v. 2.7, Stata v. 13.1, and R v. 3.3. A node was a symptom/disease and an edge represented a significantly comorbid pairwise relationship; here significance was defined as relative risk (RR) > 1, *ϕ* > 0 and *q*-value < 0.05 [15]. The *q*-value represents the local false discovery rate and was calculated to account for multiple comparisons [42]. Widths of edges represent the RR strength. The comorbidity network for the full study population is shown in figure 1. Sub-group analyses of the population are shown in figures 2–4. A description of network nodes and edges is provided in table 4. All network links and significance data are provided in electronic supplementary material, External Database S2. As a note, all reported injuries and their co-occurrence with other reported injuries or symptoms/diseases also were examined; no injuries were included in comorbidity networks because no significant associations were found.

**Table 4.** Descriptive statistics of comorbidity networks by population sub-groups. The number of people in each sub-group included individuals who reported at least one classified symptom/disease. The number of reported symptoms/diseases and possible comorbidities represent the maximum possible number of nodes and edges, respectively, for each network. The actual comorbidities represent the number of hypotheses tested for each network. There were 8001 total hypotheses tested for all comorbidity analyses (including village ecology, see electronic supplementary material), which was only 19.26% (8001/41 545) of the maximum number of hypotheses. The number of significant symptoms/diseases and comorbidities are, respectively, the actual nodes and edges in the network with RR > 1, *ϕ* > 0, RR *q*-value ≤ 0.05, and *ϕ q*-value ≤ 0.05. Using these cut-offs, 8.20% (656/8001) of all comorbidity tests were significant.

division	total people reporting at least 1 symptom/disease	reported distinct symptoms/diseases	maximum possible comorbidities	actual comorbidities (co-occurrence ≥ 1)	significant symptoms/diseases (nodes)	significant comorbidities (edges)
1. Full population						
all	14 402	80	3160	599	21	51
2. Gender (M/F) and age (years)						
F, 1–4	1113	46	1035	213	12	16
M, 1–4	1129	44	946	216	11	14
F, 5–14	2559	47	1081	263	17	26
M, 5–14	2511	55	1485	293	17	26
F, 15–49	3135	62	1891	362	19	34
M, 15–49	2899	61	1830	332	20	29
F, 50+	499	43	903	214	10	9
M, 50+	557	51	1275	230	12	11
3. Socioeconomic status index						
low	3687	62	1891	390	19	29
middle	4568	63	1953	420	20	34
high	5843	71	2485	425	23	37
4. Water, sanitation and hygiene index						
poor	4691	62	1891	388	18	27
moderate	4367	68	2278	430	20	44
high	5344	71	2485	428	20	32
5. Healthcare source (quality)						
private only (low)	3476	59	1711	395	20	45
mixed (moderate)	5069	72	2556	447	20	34
government only (high)	5857	67	2211	403	22	28

### 2.5. Network clustering and analysis

Within networks, we measured modularity (clustering of symptoms/diseases) using the algorithm described in Ahn *et al.* [43]. Edges were clustered to identify closely grouped symptoms or diseases, henceforth referred to as link clusters. This method allows for nodes to belong to multiple, overlapping link clusters. The analysis was completed in R v. 3.2.3 with the Linkcomm package [44]. Undirected networks were used with RRs as link weights. Average linkage clustering was employed. A nontrivial link cluster consisted of at least three nodes and two edges. The unsupervised approach of Ahn *et al.* [43], unlike conventional hierarchical clustering methods [45], provides a measure of partition density to optimize the cut-off for choosing the number of link clusters. Similar to modularity optimization, dendrograms showing the agglomerative cluster structure were cut at the maximum partition density. Concerning the distributional properties of networks, Python v. 2.7 with the NetworkX library [46] was used to analyse all topological properties—except link clusters—of the graphs. Network transitivity, average shortest paths, density and degree were calculated as described in Newman [47]. All symptoms/diseases with an edge assigned to at least one link cluster of a flagship symptom comprised its profile. These profiles did not necessarily include all symptoms/diseases that had an edge with a flagship symptom.

We also compared the similarity of comorbidity networks across different subsets of the study population. We calculated the overlap of nodes (symptoms/diseases) between two networks using a Jaccard index = (Network_1_ ∩ Network_2_)/(Network_1_ ∪ Network_2_). To calculate the similarity of edges between two networks, we calculated the Pearson correlation coefficient using quadratic assignment procedure (QAP) [48,49]. Edge weights were RRs. This procedure examined the presence of edges for a shared set of nodes (symptoms/diseases) between two networks. Hence, node sets differed across the compared networks for the calculation of the Jaccard index, but not in the calculation of the QAP correlation coefficient.

## 3. Results

### 3.1. Population comorbidity and flagship symptoms

A majority of the study population (88.05%; 14402/16357) reported at least one symptom/disease/injury within the three months preceding our survey (table 4). Over 60% (8718/14402) of these individuals reported two or more symptoms/diseases/injuries. Little difference was observed in the average number of symptoms/diseases/injuries (2.06–2.26) between age groups (1–4, 5–14, 15–49 and 50+ years). The average number of responses for females (2.11) and males (2.08) also was similar (*p*-value = 0.121). Most symptoms reported within our study area were computable, i.e. directly mapped to the HPO. Individuals reported a total of 144 symptoms and diseases, of which 79.17% (114) were symptoms. For all symptoms, 92.98% (106/114) were directly matched to 60 distinct HPO entries (electronic supplementary material). There were 20 categories of self-diagnoses of disease that were beyond the scope of the HPO. Although injuries were reported, injuries henceforth are not further examined because injuries had no significant comorbid conditions (electronic supplementary material, External Database S2).

Fever and diarrhoea were flagship symptoms of our study population. Fever was the most reported symptom (25.45%, 4163/16357); it is used to monitor a wide range of acute febrile diseases such as malaria and pneumonia [50]. The next most reported symptoms were chills (20.32%, 3323/16357) and diarrhoea (12.44%, 2035/16357). Chills was a ‘companion’ symptom to fever and, therefore, not a flagship symptom. Chills always was significantly comorbid (*q*-value < 0.05) with fever and had edges connected to a subset of symptoms/diseases that were associated with fever; only in few sub-group analyses did chills have one edge to a symptom/disease not comorbid with fever (e.g. low SES and poor WASH; figure 3*a*,*b*). Diarrhoea, despite also having non-communicable causes, is mainly used to monitor a wide range of prevalent viral, bacterial and parasitic gastrointestinal (GI) tract infections in low-income countries. These infections include cholera, rotaviruses, *Escherichia coli*, and (in our study area) *S. mansoni* and soil-transmitted helminths [50]. Notably, fever and diarrhoea are co-managed in integrated treatment campaigns [50]. However, diarrhoea only was associated with fever under certain demographic and social conditions. Diarrhoea and fever were correlated (*q*-value < 0.05) for females aged 1–4 and 15–49 years (figure 2), individuals within households with poor WASH behaviours (figure 3*b*), and individuals belonging to households that seek medical care from private clinics (figure 4). Unlike fever, diarrhoea did not have comorbid conditions within all age groups, i.e. diarrhoea was not always a node in the comorbidity networks. The elderly (aged 50+; electronic supplementary material, table S2) did not display any significantly comorbid (*q*-value < 0.05) symptoms/diseases with diarrhoea (figure 2).

### 3.2. Flagship symptom targeting

Fever was a structurally important high degree node (figures 2–4) [8]. Fever had an average of 6.824 (range 3–10, s.d. 2.215) edges while diarrhoea had only an average 2.294 edges (range 0–4, s.d. 1.312). Hence, the most prevalent symptoms/diseases were not necessarily equivalent to the symptoms/diseases with the greatest range of comorbid conditions. Poor appetite was not a flagship symptom and had an average of 6.059 edges (range 3–11, s.d. 2.384). Malaria was most often reported, but in a majority of networks (15/17) it had only two significant (*q*-value < 0.05) connections to symptoms of fever and chills and at most three edges (cough or weight loss). Not all frequently reported symptoms/diseases were comorbid. Abdominal pain, which was reported by 7.18% (1034/14402) of people experiencing at least one symptom/disease, was not significantly correlated with any other symptoms/diseases (*q*-value > 0.05). Flagship symptoms had mostly weak associations (avg. fever RR 1.641, s.d. 0.409; avg. diarrhoea RR 1.765, s.d. 0.466) with other symptoms/diseases. The highest RR of comorbidity was predominately between symptoms/diseases on the periphery of the networks (figures 1–4). Thus, targeting the most likely comorbidities would only reduce the size of the networks. Addressing the causes of symptoms with many weak associations including fever—in all demographic, SES, and WASH contexts—is an efficient treatment strategy for breaking the networks and consequently reducing many, diverse comorbidities.

### 3.3. Biosocial determinants of comorbidity structure

Flagship symptoms were situated in diverse comorbidity networks with varying node types and structures. Demographic characteristics (figure 2), SES (figure 3*a*), WASH behaviours (figure 3*b*) and the type of medical care (figure 4) each influenced the comorbidities observed. Despite only 35 symptoms/diseases of 80 reported symptoms/diseases/injuries ever being significantly comorbid (*q*-value < 0.05, table 4 and electronic supplementary material, table S1), no two comorbidity networks in the sub-group analyses shared the exact same node set (table 5). The overlap of nodes was highest between individuals aged 5–14 and 15–49 for both females (56.5%) and males (76.2%). Some shared symptoms/diseases included sexually transmitted infections (STIs), itchy rash (pruritus) and GI haemorrhage. Children aged 1–4 years had few symptoms/diseases in common with elderly individuals aged 50+ years. Only 37.5% and 35.3% of nodes overlapped between these age groups for both females and males, respectively. For example, seizures and diarrhoea was reported only for young children, whereas difficulty seeing and walking was noted only in the elderly group. When individuals in the same age group were compared by gender, surprisingly, females and males aged 15–49 were the most similar with 77.3% shared symptoms/diseases. This result suggests that, despite the onset of biological differences in reproductive health, comorbidities in women and men of reproductive age might be best co-targeted rather than treated as entirely distinct in our study area. Elderly females and males were the most dissimilar with respect to symptoms/diseases reported (57.1% overlap). Hence, no convergence in comorbidity was observed at the end of life.

**Table 5.** Network overlap between sub-groups of the study population. Most of the comparisons between two networks (20/27) showed a significant (*p*-value < 0.05) positive correlation between edge sets that varied in strength from *r* = 0.008 to *r* = 0.899. Across all sub-group analyses, the coefficient of correlation (magnitude) between edge sets for two networks was negatively associated (*r* = −0.414, *p*-value = 0.0312) with the number of shared nodes. Thus, the higher the node overlap was for two networks then the more likely it was that the strength and presence of the edges between those shared nodes differed.

Network_1_	Network_2_	Jaccard index	QAP Pearson corr. coeff.	QAP *p*-value
F, 1–4	F, 5–14	0.526	0.235	0.035
F, 1–4	F, 15–49	0.550	0.078	0.177
F, 1–4	F, 50+	0.375	0.888	0.002
F, 5–14	F, 15–49	0.565	0.853	<0.001
F, 5–14	F, 50+	0.421	0.741	<0.001
F, 15–49	F, 50+	0.526	0.113	0.118
M, 1–4	M, 5–14	0.647	0.714	<0.001
M, 1–4	M, 15–49	0.550	0.301	0.010
M, 1–4	M, 50+	0.353	0.899	0.002
M, 5–14	M, 15–49	0.762	0.149	0.033
M, 5–14	M, 50+	0.611	0.092	0.094
M, 15–49	M, 50+	0.524	0.109	0.078
F, 1–4	M, 1–4	0.643	0.863	<0.001
F, 5–14	M, 5–14	0.700	0.340	0.004
F, 15–49	M, 15–49	0.773	0.108	0.042
F, 50+	M, 50+	0.571	0.093	0.161
low SES	middle SES	0.773	0.397	0.004
low SES	high SES	0.615	0.411	<0.001
middle SES	high SES	0.792	0.151	0.018
poor WASH	moderate WASH	0.727	0.351	0.003
poor WASH	high WASH	0.727	0.081	0.093
moderate WASH	high WASH	0.739	0.050	0.097
government health centres	mixed care	0.615	0.565	<0.001
government health centres	private clinics	0.615	0.565	<0.001
mixed care	private clinics	0.818	0.008	0.153
low SES	poor WASH	0.762	0.808	<0.001
high SES	high WASH	0.720	0.554	<0.001

Young children and individuals in worse-off households had flagship symptoms within network structures that displayed the highest comorbidity burden and/or were conducive to the onset of new comorbidities from existing flagship symptoms. Network density and average shortest path are indirect measures of comorbidity burden (electronic supplementary material, figure S9). Density was indicative of the maximum possible edges (comorbidities) that can exist among a set of symptoms/diseases. Average shortest paths represent the distance, i.e. number of steps/edges, between different symptoms/diseases. Diseases have been shown to develop new comorbidities with other diseases that are close with respect to network distance [15,16]. Females (0.242) and males (0.255) aged 1–4 years had the highest density of edges across age. Density increased as healthcare quality decreased (0.121 to 0.237). Individuals in low/poor SES, WASH, and medical care households had a smaller average shortest path compared to individuals in high SES, WASH, and medical care households (electronic supplementary material, figure S9).

### 3.4. Correlates of the type of healthcare accessed

Age, gender, SES, WASH, and the type of healthcare accessed are characteristics that do not exist in isolation. As we are concerned with health-seeking behaviours, we examined how age, gender, SES, and WASH were associated with the type of healthcare accessed. The type/severity/onset of symptoms/diseases may also affect the type of health accessed; however, this potentially endogenous relationship cannot be studied from the cross-sectional analysis conducted here and is beyond the scope of this study.

The predictors of the type of healthcare accessed are shown in table 6. Individuals in low or middle SES households were, respectively, 1.775 and 1.445 times more likely to use private/informal healthcare than government healthcare when compared to individuals in high SES households (*p*-value < 0.01). Interestingly, individuals belonging to households with poor WASH behaviours were less likely (odds ratio 0.574) to use private/informal healthcare and thus more likely to use government healthcare when compared to individuals within households with high WASH behaviours (*p*-value < 0.001). Males of reproductive age (15–49 years) were 1.211 times more likely (*p*-value = 0.030) to use private/informal healthcare than government healthcare when compared to females aged 1–4 years and when controlling for all other age and gender groups. Further research is needed to ascertain whether the aforementioned associations are good proxy indicators of other influences on the type of healthcare accessed. Other influences may include the severity of perceived illnesses with age, affordability of healthcare, and individual preferences for medical care.

**Table 6.** Predictors of the type of healthcare accessed. AUC = area under receiver operating characteristic curve. SES = socioeconomic status. WASH = water, sanitation and hygiene. A multinomial logistic regression is shown with robust standard errors clustered by household to account for intrahousehold correlation.

	odds ratio	s.e. clustered by household	*p*-value	95% CI	
outcome = only private clinics accessed^a^					
female, 5–14 years^b^	0.962	0.094	0.693	0.795	1.164
female, 15–49 years^b^	0.976	0.080	0.770	0.832	1.146
female, 50+ years^b^	0.888	0.129	0.414	0.668	1.181
male, 1–4 years^b^	1.098	0.122	0.399	0.884	1.365
male, 5–14 years^b^	0.993	0.098	0.944	0.819	1.205
male, 15–49 years^b^	1.211	0.106	0.030	1.019	1.438
male, 50+ years^b^	0.956	0.132	0.745	0.730	1.253
low SES^c^	1.775	0.227	<0.001	1.382	2.280
middle SES^c^	1.445	0.186	0.004	1.123	1.860
poor WASH^d^	0.574	0.072	<0.001	0.449	0.733
moderate WASH^d^	1.059	0.134	0.649	0.826	1.358
constant	0.513	0.070	<0.001	0.393	0.669
outcome = mixed care: government health centres and private clinics accessed^a^					
female, 5–14 years^b^	0.938	0.080	0.454	0.793	1.109
female, 15–49 years^b^	0.871	0.065	0.065	0.753	1.008
female, 50+ years^b^	0.882	0.112	0.323	0.687	1.132
male, 1–4 years^b^	1.032	0.103	0.749	0.849	1.255
male, 5–14 years^b^	0.900	0.075	0.209	0.764	1.060
male, 15–49 years^b^	0.930	0.073	0.355	0.796	1.085
male, 50+ years^b^	0.878	0.103	0.268	0.697	1.106
low SES^c^	1.016	0.118	0.894	0.808	1.276
middle SES^c^	0.882	0.100	0.271	0.706	1.103
poor WASH^d^	0.597	0.068	<0.001	0.477	0.747
moderate WASH^d^	1.266	0.150	0.046	1.004	1.596
constant	1.067	0.128	0.587	0.844	1.350
*obs.* (individuals)	14 098				
*obs.* (households)	3342				
*Wald statistic*	117.89	*p*-value < 0.0001			
*AUC*	0.9906				
*mean AUC; 10-fold cross-validated*	0.9558				

^a^Base outcome = only government health centres accessed.

^b^Base category = females, 1–4 years.

^c^Base category = high SES.

^d^Base category = high WASH.

### 3.5. Flagship symptom profiles and self-misdiagnoses

Depending on biological (figure 2) and social contexts (figures 3 and 4), fever and diarrhoea, respectively, had 1–5 and 0–1 profiles. Though beyond the scope of this paper, diarrhoea was grouped in three profiles in villages with high *S. mansoni* and moderate hookworm prevalence (electronic supplementary material, figure S8). Henceforth, we focus on the discussion of fever due to its profile variation and known difficulties of diagnosis [28]. Each fever profile was indicative of a densely connected group of similar comorbidities. Thus, each fever profile, if clinically verified and formally diagnosed, might be associated with a different underlying cause. The number of fever profiles was highest in females aged 5–49 and males aged 15–49 (three profiles), individuals who accessed private clinics (four profiles), high SES households (four profiles), and people with moderate WASH behaviours (five profiles). Fever afflicting these sub-groups may be most difficult to diagnose due to a wide range of potential causes and may therefore require intensified monitoring in comparison to population sub-groups with fewer fever profiles.

Actual symptom/disease differences between flagship symptom profiles may provide an overview of how perceived health status varies within a rural poor community. This rapid screening of symptoms/diseases may help refine a shortlist of potential causes and the required instruments for later clinical diagnoses of flagship symptoms by population sub-group. This shortlist, albeit a patient-directed screening of illness, is needed in areas where resources are unavailable to clinically examine all individuals for all potential causes of all symptoms/diseases. Focusing on the main profiles of fever, where at least two edges are grouped, fever had only 1–2 profiles. Two main fever profiles were observed in the full study population (figure 1). The first profile consisted of chills, seizures, vomiting and weight loss while the second profile comprised chills, STIs, cough, poor appetite and pruritus. If these two profiles were applied to choose diagnostic tools to clinically verify the causes of fever for all individuals in the study villages then age-related differences would be masked (figure 2). With the addition/subtraction of a few variable symptoms/diseases, the first profile found in the full population appears in most sub-group analyses (figures 2–4). However, a fever profile including STIs/pruritus only was observed in individuals aged 15–49 years. Low and middle SES appears to contribute to the onset of a fever profile including STIs/pruritus (figure 3*a*). Interestingly, variation in hygienic behaviours (WASH) did not appear to have an influence on the inclusion of STIs/pruritus in a main fever profile; STIs/pruritus was observed in main fever profiles for all WASH categories (figure 3*b*). When treating febrile illnesses for females and males of reproductive age in our study area, screening for STIs may be needed in addition to the standard tracking of pneumonia and malaria [50].

Fever profiles, and hence network modularity, may reveal fever self-misdiagnoses. The size of fever profiles varied from 3 to 11 symptoms/diseases (figures 2–4). The wide range of symptoms/diseases associated with fever may explain why self-reported diagnoses of the underlying causes of fever are often inaccurate [28,51]. This problem concerns patients assuming fever is only indicative of malaria [28]. Our data support this inference. As previously noted, malaria always was significantly associated with fever and chills (*q*-value < 0.05). If individuals were prompted to note the symptoms/diseases associated with fever instead of using an unguided approach as employed here then we might expect individuals to only group fever with malaria. However, malaria was not consistently in a fever profile. Malaria was not in any fever profiles for children aged 1–4 years, males aged 5–14, females aged 15–49, and for individuals within households with moderate WASH behaviours or households that access only government health centres or only private clinics (figures 2–4). Notably, individuals within households that accessed private clinics had two main fever profiles and no fever profiles that included malaria (figure 4).

## 4. Discussion

Here we showed how data-driven tools from network medicine could inform precision global health interventions. We found that the symptoms prioritized by a majority of individuals, here fever and diarrhoea, accorded with the symptoms associated with high rates of morbidity and mortality in low-income countries [52]. These flagship symptoms used for disease surveillance were embedded in comorbidity networks that varied depending on age, gender, SES, WASH, and type of medical care.

For fever, the analysis of this variation revealed biosocial variation of comorbid symptoms/diseases, potentially what diagnostics to consider by population sub-group, and potential self-reported misdiagnoses/mistreatments. Fever was commonly reported within all age groups and in its profile STIs were included for individuals of reproductive age and within households of low to middle SES. Though fever always was significantly co-reported with malaria, it was not always grouped within fever profiles. Thus, we question whether malaria was always the cause of fever. This information is important for developing accurate diagnostic strategies for fever in our study region where a majority of individuals display positive malaria parasitaemia [53,54]. Confirmatory diagnoses where a rapid diagnostic test (RDT) is conducted after the detection of fever and then a positive RDT result is used to attribute fever to malaria may detract from the detection of the true causes of fever [1]. In Uganda, fever is treated using home-based management strategies where community health workers distribute a package of drugs to treat malaria, diarrhoea and pneumonia in all febrile children less than 5 years of age [55]. Yet, fever was central in the comorbidity networks for all age groups. Targeting the causes of fever would likely reduce a majority of the comorbidities in all age groups of our study population. Home-based management strategies, if expanded to all age groups, might consider screening for STIs as another potential cause of fever for individuals aged 15–49 years.

Profiling flagship symptoms using network analysis provides one method of navigating a complex symptom/disease landscape to improve fever monitoring and diagnostic strategies in rural poor communities. Individuals who only used private clinics were most likely to have multiple profiles, and therefore potentially multiple underlying causes of fever and least likely to have fever associated with self-reported malaria when compared to individuals who used government health centres. Yet, individuals who use private clinics are most likely to mistreat fever [28]. In our study area, individuals have been shown to receive first aid for fever by accessing private clinics to purchase antimalarials, without clinical diagnoses, to mistreat pneumonia as malaria [28]. Private clinics could be targeted/incentivized to encourage referrals of complex cases of fever to government health centres.

We showed that data-driven tools from network medicine could be applied in low-income settings without EMRs. Easy-to-collect phenotypic data, here self-reported symptoms, were mapped to an internationally recognized medical classification, the HPO [40]. This mapping made computable heterogeneous descriptions of illness. We studied a geographical area already identified through one method of precision global health, i.e. geotargeting of malaria and schistosomiasis [25]. Accordingly, our results may be applicable to other similarly identified areas. An unguided illness prompt could be added to interventions utilizing geotargeting to better target individuals. For more widespread application, the illness prompt used here may be incorporated into Demographic and Health Surveys (https://dhsprogram.com, accessed 9 October 2018). Our method is not limited to small, rural communities. Hospitals with limited EMRs can use the recently released HPO Patient Archive Software [40] to elicit and categorize symptoms for a large number of patients.

We demonstrated how to use network-based approaches to validate self-reported symptoms. Network grouping of self-reported symptoms revealed clusters of symptoms that were consistent with, and indicative of, symptoms/diseases previously clinically observed in the study area [28,50,53,54]. This network validation demonstrates the potential utility of large-scale self-reported symptom collection in a public health context. Large-scale collection of self-reported symptoms will contain important information for simplifying diagnostic and treatment strategies. The data-driven approach shown in this study enables a first ‘scan’ of morbidity in a population that may be used to refine the selection of clinical diagnostic tools and to construct a shortlist of biosocial factors expected to confound clinical diagnosis. In addition to presenting a novel method to validate self-reported symptoms, we also employed multiple conventional methods. As previously noted, prevalence of self-reported flagship (common) symptoms accorded with published literature using clinically verified outcomes. Moreover, in our study villages, self-reported malaria (53.90%, 8816/16357) was similar in magnitude to the prevalence (40–60%) previously measured with microscopy in other studies [53,54]. This result suggests that self-reported diagnoses might help guide prospective identification of at-risk villages for malaria during presumptive treatment and bed net campaigns. Clinical review also was conducted to check the agreement of self-reported symptoms with expert medical opinion. The supervisors of our lay village surveyors were medical professionals from the Ministry of Health, who helped to check, though not alter, incoming field data. The overall field supervisor was the Assistant Health Commissioner of Uganda. The nurse members of our field supervisors provided feedback in daily meetings, as did our experienced team of qualified parasitology technicians. Reported symptoms were translated and reviewed by medical professionals from the Ugandan Ministry of Health as being consistent with medical diagnoses, based on expert medical examination as previously observed in the study area. Lastly, by mapping self-reported symptoms to the HPO, we consolidated lay reports and matched them to internationally recognized types of symptoms.

There are limitations to the use of self-reports that can be addressed in future work. To minimize recall bias, we did not elicit the order that symptoms/diseases were experienced. Thus, directionality in the RR could not be established. Although a cross-sectional design sufficed here for the examination of biosocial variation, any studies interested in the progression of self-reported symptoms/diseases over time should consider establishing a participant cohort with repeated surveys. We also were interested in patient-prioritized symptoms/diseases to identify the illnesses for which individuals are most likely to seek treatment. Consequently, underreporting may have occurred. Latent symptoms, i.e. symptoms not observed by the patient, and conditions that are not viewed as a health problem, e.g. possibly maternal or mental health issues in our study communities, might be underreported. Any sensitive or stigmatizing information, such as a patient having had past clinical exams confirming HIV status or possible conditions associated with HIV infection risk, also may be underreported. If mental health, maternal health and HIV risk were not underreported then future studies should consider examining a larger patient cohort than used here to power the analysis of these rarely reported medical conditions (electronic supplementary material, External Database S1). Concerning health-seeking behaviours, a limitation of this study was that we did not have information about the frequency of healthcare accessed or informal costs, e.g. travel costs of accessing private versus government health services. The ranking of self-reported comorbidities by severity was beyond the scope of this study. However, future research may develop a severity ranking/index for self-reported comorbidities to examine whether such severity influences the type of healthcare accessed.

If the self-reported symptoms/diseases are followed up with clinical testing and the analyses are repeated in other low-income countries then network medicine may inform the design of precision global health interventions for complex medical syndromes. From the perspective of the participant, we showed how individuals value/rank health problems, what was likely to be reported, what was likely to be misreported/self-misdiagnosed, and how the aforementioned issues were influenced by demographic, social, and behavioural factors. This information is crucial for classifying patients who may be less likely to seek medical care or whose environmental/lifestyle factors may interfere with accurate clinical diagnosis or treatment. Our analysis also demonstrated how the general properties of complex networks reveal the magnitude and organization/structure of comorbidity burdens. We focused on fever as its recognition by patients or lay health workers is used to guide malaria and diarrhoea treatment, to monitor infection outbreaks, and to refer individuals to clinically trained professionals for formal diagnosis. Network medicine provides a set of powerful analytical tools that may lead to improved diagnostic/treatment strategies that are tailored to the biosocial variation of disease.

## 5. Conclusion

We showed that individuals who have low socioeconomic status and poor hygienic/sanitary behaviours, and who receive poor quality medical care display the greatest self-reported comorbidity burden with the highest density and variability of comorbid conditions. The social confounders of patient-recognized morbidity have been neglected in data-driven, unsupervised epidemiological studies. And, to our knowledge, no data-driven or network analyses of patient symptoms have been conducted in low-income countries. This study revealed that self-reported morbidity varies widely, dependent on personal demographic and socioeconomic context. We demonstrated that comorbidity network structures can be used to track self-misdiagnoses of flagship symptoms, such as fever, that are used to monitor diseases of global health concern. If implemented prior to clinical diagnoses and drug administration, the powerful analytical approach proposed here may inform medical practices concerning public health. Data-driven network analyses of self-reported symptoms can identify a shortlist of potentially difficult-to-diagnose symptoms by population sub-group. We anticipate that our network-based approach for analysing self-reported symptoms will contribute to the more efficient use of community-based treatment and clinical facilities/expertise in resource-limited settings.

## Supplementary Material

Supplementary Material

## Supplementary Material

External Database S1

## Supplementary Material

External Database S2

## Data accessibility

All relevant data are included in the paper and electronic supplementary material files.
