# Supplementary material for: Precision global health and comorbidity: a population-based study of 16 357 people in rural Uganda
Source: J R Soc Interface. 2018 Oct 31;15(147):20180248. doi: 10.1098/rsif.2018.0248 (PMC6228477; doi:10.1098/rsif.2018.0248)
Supplement: Supplementary Material [file rsif20180248supp1.pdf]

Supplementary Information for "Precision global health and comorbidity: a population-based study of 16,357 people in rural Uganda"

Goylette F. Chami*, Narcis B. Kabatereine, Edridah M. Tukahebwa, David W. Dunne

Table of Contents

Materials and methods 2
Figure S1: Mapping of reported symptoms/diseases to the human phenotype ontology (HPO) 9
Figure S2: Directed acyclic graph, force-directed layout, of HPO for reported symptoms 10
Figure S3: Directed acyclic graph, hierarchical layout, of HPO for reported symptoms 11
Figure S4: P-value distributions and estimated proportion of true null hypotheses for q-values. 12
Figure S5: Descriptive statistics for relative risk q-values. 13
Figure S6: Descriptive statistics for Spearman ρ q-values. 14
Figure S7: Principal component analysis scores from first component. 15
Figure S8: Comorbidity networks by village ecology. 16
Figure S9: Trends in global network density and avg. shortest path. 17
Table S1: Descriptive statistics of ecological sub-group of comorbidity network analyses 18
Table S2: Socioeconomic variable weights from first principal component 18
Table S3: Water, sanitation, and hygiene (WASH) variable weights from first principal component 19
Table S4: Village characteristics by intestinal helminth prevalence 19
References 20

Materials and methods

Household survey

In November 2013, data was collected for 16,357 individuals in 3,491 households in 17 villages of Mayuge District, Uganda. The household head (and/or wife) was interviewed in the local language of Lusoga and information was directly entered on Google Nexus 7 tablets using Open Data Kit[1] for the survey design. The respondent provided medical and socioeconomic information for all members, including themselves, aged 1+ years in the household.

Symptom/disease reporting

The household head (and/or wife) reported the symptoms/diseases experienced by all members of the household within the three months preceding the survey. The reported symptoms/diseases were provided as a sequence of symptoms and/or diseases, i.e. in list form as opposed to open natural text descriptions. In August 2016, a medical professional based at the Ministry of Health in Kampala, Uganda translated the list of all diseases and symptoms from Lusoga to English. Only literal translations of each term were provided; there were no translations that combined any symptoms to provide a diagnosis.

Human Phenotype Ontology mapping

The Human Phenotype Ontology (HPO) is a classification of clinical phenotype abnormalities [2]. Though originally designed for Mendelian and rare disorders, the ontology was expanded in 2016 to include many symptoms of common diseases. HPO exhibits many benefits not provided from the ICD10-CM classification (https://www.cms.gov/Medicare/Coding/ICD10/index.html Accessed Mar. 28, 2018), which is designed for billing purposes. The HPO focuses on the patient as opposed to the disease as the subject of interest. HPO terms are not explicitly related to concepts from human physiology and laymen terms are included as synonyms.

The reported symptoms were matched to the HPO using the March 9, 2017 release (http://purl.obolibrary.org/obo/hp.obo). The matching data is provided in External Database S1. Figure S1 shows the number of reported symptoms/diseases and the matching to the HPO. There were 144 terms used to describe reported symptoms/diseases; 114 were symptoms, 30 were diagnoses that are beyond the scope of the HPO, and 8/114 symptoms were too vague to be matched to one entry in the HPO. One of the 8 unmatched symptoms, generalized pain, was in the broad entry of 'pain' in the HPO. However, since the entry of 'pain' would have engulfed many more specific symptoms, e.g. abdominal pain, it was not coded at all. Only 6/14459 people reporting at least one symptom had reported generalized pain. The 8 vague symptoms were not further analyzed. After dropping the 8 vague symptoms, the number of people reporting at least 1 symptom/disease was reduced from 14,459 to 14,402. The 30 reported diagnoses were manually grouped into 20 categories. For example, reported 'malaria' and 'cerebral malaria' were grouped as 'malaria'. One diagnosis, broken hand, was within the HPO as 'broken hand bones' but was not assigned an HPO id because 'broken hand' could not be distinguished from other reports of general accidents. Instead, 'broken hand' was manually coded into the injury/trauma category.

Matching consisted of assigning one HPO primary id to each of the 106 symptom terms. First, the HPO primary id was assigned manually by finding the HPO entry term, synonym, or description that contained the reported symptom. Redundant symptoms, i.e. slight variations in

reporting of the same symptom, were manually assigned to the same HPO primary id, e.g. ‘eye problem’ and ‘eye disease’ was assigned to ‘abnormality of the eye’, id HP: 0000478. We assigned 86 unique HPO primary ids to the 106 symptoms.

Second, we ensured that each assigned HPO primary id was distinct from another assigned HPO primary id. To be distinct, the manually assigned HPO primary id could not be a subclass of another assigned HPO primary id. These nested reported symptoms occurred when different levels of detail were provided about a similar problem. For example, ‘ear pain’ and ‘ear problem’ were both reported. The latter was classified as an ‘abnormality of the ear’ and assigned to HP:0000598; it is also a superclass of the HPO term ‘ear pain’, id HP:0030766. Formally, the full HPO text was parsed using Python v2.7 (www.python.org) so that a directed edge was generated between each primary HPO term and their direct superclasses. This procedure generated one directed acyclic graph (DAG), which is the design of the HPO, with 211 nodes and 242 edges and the root as HPO term ‘All’, id HP:0000001 (External Database S1). The DAG is presented in Figure S2 and it is shown again in Figure S3 with a hierarchical layout. These figures were generated using Cytoscape v3.3.0 [3]. Using the NetworkX library in Python[4], we iterated over each of the matched 86 HPO primary ids to find all ancestors (parents) in the DAG. The ancestors were then searched to find any overlap with the other 85 HPO primary ids. This search revealed that 27 of the 86 manually assigned HPO primary ids were descendants (children) of another assigned HPO primary id in the DAG. Four of the 27 nested HPO primary ids had two ancestors. Of these terms, 3 of 4 were straightforward choices amongst the two parents; one parent was a superclass of the other parent. For 1 of the 4, a choice was made to classify ‘gastrointestinal hemorrhage’ as an ‘abnormality of the digestive system’ as opposed to an ‘abnormality of the cardiovascular system’. This choice was due to the known morbidity attributable to intestinal helminth infections in the study villages [5]. This classification also resulted in 1 of the 27 HPO primary ids no longer being a subclass of another assigned HPO primary id. For each manually assigned HPO primary id that was a subclass of another manually assigned HPO id, the superclass HPO id was used in place of the HPO primary id. Ultimately, there were 60 unique and distinct HPO ids assigned (86 original - 26 nested ids) to 106 reported symptoms. Together with the 20 diagnosis groupings, there were 80 total diagnoses and symptoms in which to explore comorbidity relationships. With these groupings, if an individual listed two symptoms and one of those symptoms was a subclass of another symptom, e.g. ‘ear pain’ is a subclass of ‘ear problem’ (HPO term ‘abnormality of the ear’), then these two symptoms would be counted as one reported symptom.

Comorbidity network construction

All comorbidity networks consisted of a maximum of 80 nodes, corresponding to the 60 symptoms mapped with the HPO and 20 disease categories. Accordingly, for each network, there was a maximum of 3,160 pairwise combinations (edges) of the 80 symptoms/diseases. In the sub-group analyses (see next section), some symptoms/diseases may not be present in the sub-group of interest. Isolates are not presented. Undirected network edges were generated in Stata v13.1 (*StataCorp LLC*) as follows. If two symptoms/diseases were reported together in the population more often than expected by chance then an edge was generated between those symptoms/diseases. Hence, only symptoms/diseases with a ≥1 coocurrence with at least one other symptom/disease were investigated in the correlation analyses. Relative risk (RR) [6] was used to determine the edge strength. Fisher’s exact 2-sided p-value was calculated [7-9]. Though

relative risk tends to overestimate rare events, Fisher's exact p-value is conservative for rare events [10]. RR 95% confidence intervals and Z-statistics were calculated as described in Katz [11] and Altman [12], respectively. We also calculated the ϕ correlation, its 95% confidence interval, and approximate p-value [6, 8]. The ϕ correlation, with possible values between [-1,1], is a complementary measure to RR, but ϕ underestimates associations between two symptoms/diseases of vastly different prevalence [6, 8].

An edge was generated between two symptoms/diseases if the RR>1, ϕ>0, RR q-value ≤0.05, and ϕ q-value ≤0.05. The q-value is a measure of the false discovery rate (FDR) and a correction for multiple comparisons. The FDR is a directly interpretable measure of the percentage of false positives amongst our significant results and is less prone to researcher bias than the Bonferroni correction (family-wise error rate)[13]. For each network, we estimated the q-values as described in Storey and Tibshirani [14] and using the Qvalue package in R v.3.2.3 [14, 15]. Specifically, the q-value extends the Benjamini-Hochberg FDR correction [16] to fully automate the estimation of the number of truly null hypotheses and to convey the percentage of significant findings that may be false positives around a particular statistical test of interest. There were 8,001 correlations run in total to investigate comorbidity associations; the q-values were calculated using the results from all tests (Figures S4-S6).

Comorbidity networks of population sub-groups
Six divisions in the study population were examined to understand how comorbidity structure varies by social and environmental contexts [17]. The division types and definitions are provided below. For the descriptive statistics of each division, refer to Table 4 and Table S1. All network statistics are provided in External Database S2.

1. Full population
2. Gender and age
    a. Female, 1-4 years
    b. Male, 1-4 years
    c. Female, 5-14 years
    d. Male, 5-14 years
    e. Female, 15-49 years
    f. Male, 15-49 years
    g. Female, 50+
    h. Male, 50+
3. Socioeconomic status (SES) index
    a. Low
    b. Middle
    c. High
4. Water, sanitation, and hygiene (WASH) index
    a. Poor
    b. Moderate
    c. High
5. Health care source (quality)
    a. Private only (low)
    b. Mixed (moderate)
    c. Government only (high)

6. Village intestinal helminth prevalence
    a. High *Schistosoma mansoni* (*S. mansoni*), High hookworm
    b. High *S. mansoni*, Moderate hookworm
    c. High *S. mansoni*, Low hookworm
    d. Moderate *S. mansoni*, High hookworm
    e. Moderate *S. mansoni*, Moderate hookworm

1. Full population. We provided the comorbidity structure of all individuals in the study area as an overall summary of community health and reference category for population sub-group comparisons. This information is useful for untargeted, *en masse* health interventions. For example, mass drug administration (MDA) is an *en masse* treatment programme that provides preventive chemotherapies to treat populations at risk of neglected tropical diseases (NTDs), including schistosomiasis and soil-transmitted helminths [18]. Information about the comorbidities affecting the population of interest can be used to design integrated MDA programmes, e.g. the development of treatment packages for comorbid NTDs [19].

2. Gender and age. This sub-group analysis provides a cross-sectional view of disease onset and progression across life [20] in a rural sub-Saharan African (SSA) population. Four age groups (1-4, 5-14, 15-49, and 50+) were analyzed against gender. The percentage of the study population in each gender-age group is provided in Table 1. The age cutoffs were chosen to represent groups of international importance. In low-income countries, mortality risk is highest amongst children less than 5 years of age [21]. The under-5 mortality rate is highest in SSA [21]. The age group of 5-14 years captures school-aged children, who are the focus of MDA programmes, and the population subset with the heaviest infection intensities of schistosomiasis [5, 22]. Individuals aged 15-49 represent the population of reproductive and working age [21, 23]. Hence, the comorbidity of females in this group is a profile of risks affecting maternal health. With only 7.19% of individuals aged 50+ years (Table 1), this category represents the elderly in our study population. It also is one age group used to calculate global life expectancy from the Global Burden of Disease Study [21].

3. Socioeconomic (SES) index. SES is a direct measurement of health inequalities, which affect the lifetime risk of disease and access to resources for medical care [24]. Globally, mortality is highest amongst the poorest of the poor [24]. For NTDs, socioeconomic status is a key determinant of the risk of infection and access to medicines through MDA [5, 25].

Amongst all households 36.44% (1272/3491) were farming households only, 21.23% (741/3491) were fishing households only, and 8.14% (284/3491) of households had income-earning individuals with both occupations. The majority tribe variable indicates if a household belongs to the majority tribe in their village and district (here, Musoga). The number of years the household was settled in the village, home ownership (binary), and home electricity (binary) also were measured. There were 66 households (318 people) missing both religion and tribe information, and 5 households (17 people) with missing information on the number of years in the village. Due to this missing information, a total of 2% (70/3491) households and 2.02% (331/16357) of people were excluded from the construction of the SES index.

To reduce dimensionality and to account for any confounding (correlation) amongst SES variables, principal components analysis (PCA) was used to develop a SES index [26, 27]. Specifically, polychoric PCA was used to account for the several binary SES indicators and was run at the household level in Stata v13.1 (*StataCorp LLC*) [28]. The first 3 eigenvalues were 2.35, 1.67, and 1.19 and explained, respectively, 21.38%, 15.14%, and 10.86% of variation. The variation explained by the first component is in the standard range for SES indices [26, 27, 29]. The SES score for each household was generated from the first component and assigned to all members of the household. All SES variables entered in the direction as expected for the first component (Table S2). The highest weights were assigned to council membership (0.73), other status (0.71), and home ownership (if renter, weight=-0.65). SES scores were normally distributed across households (Figure S7). Accordingly, SES scores were split into 3 categories to develop an ordinal index of low (SES score cutoff=-0.47, 33.33^{th} %ile), middle (SES score cutoff=0.33, 66.67^{th} %ile), and high SES. The 3-category SES index was internally coherent [27]; a summary of the mean value of each SES variable by SES category is provided in Table 2.

4. Water, sanitation, and hygiene (WASH) index. WASH may arguably be the most widely advocated health intervention for low-income countries [30]. The promotion of WASH is essential to achieving many of the United Nation's Global Goals—a set of development targets—for the year 2030 [31]. For NTDs, WASH is a critical component in efforts to reduce pathogen transmission[32] (http://washntds.org). Many NTDs persist due to the lack of access to safe water and sanitation [32]. The components of WASH are highly interdependent. We developed a WASH index to account for these interdependencies and to measure the quality of WASH engagement.

Tetrachoric PCA with the seven binary WASH indicators was run in Stata v.13.1 (*StataCorp LLC)* [28]. The first component explained a remarkable 41.97% of variation (2.94 eigenvalue) in WASH. The second and third components explained 18.67% and 13.70% of variation with 1.31 and 0.96 eigenvalues, respectively. WASH scores were generated at the household level from the first principal component and assigned to all members of the household. The scores were roughly uniformly distributed across households (Figure S7) with no clear evidence of clumping [27]. Accordingly, WASH scores were split into 3 categories to develop an ordinal index of poor (WASH score cutoff=-0.63, 33.33^{th} %ile), moderate (WASH score cutoff=0.10, 66.67^{th} %ile), and high WASH quality.

Interestingly, all WASH indicators did not enter as expected in the first principal component (Table S3). All variables used were, as dictated by the WHO/UNICEF JMP definitions, positive indicators of WASH quality. The variables that entered as expected were improved drinking water (0.25), improved cooking water (0.67), improved bathing water (0.80), water availability/quantity (0.08), and improved sanitation (0.19); the weights for when each variable =1 are listed in parentheses. Positive values (=1) for water treatment (-0.18 weight) and soap availability (-0.30 weight) did not have a positive contribution to the WASH quality index. This negative contribution was likely due to crowding out of WASH behaviours. We discovered that multiple WASH behaviours might produce a crowding out effect, i.e. when a household engaged in one WASH behaviour then a complementary behaviour might have been perceived as not necessary. In our study population, this crowding out effect occurred with a trade off between improved sanitation and hygiene/soap availability as well as water treatment and improved

drinking water. Water treatment was negatively associated with the use of improved drinking water sources (Spearman ρ -0.20, p-value<0.0001, obs. 3491). Soap availability was negatively correlated with improved sanitation (Spearman ρ -0.12, p-value<0.0001, obs. 3491). Future studies should further investigate possible crowding out effects as an unintended consequence of promoting multiple complementary WASH interventions. A summary of the mean value of each WASH indicator by WASH category is provided in Table 3.

5. Health care source (quality). To understand how the type of care affects comorbidity, we asked households to list where they "usually go to get drugs and other medical care." The household response was assigned to all members of the home. An index was developed to measure the source and quality of care. Two sources were possible: private and government. Private care was indicative of more informal, unregulated care and included private clinics, drug shops, herbalists or witch doctors, and other unspecified options not noted in government care. Government care measured formal, regulated medical care and included government health centres and village health teams. Households could list multiple sources. Accordingly, three categories were developed for the health care index: private only, mixed source, and government only. These categories, respectively, are thought to capture low, moderate, and high quality medical services. Private care was viewed as suboptimal to government care because individuals in our study area[33] and in other SSA countries, e.g. Kenya[34], are able to purchase drugs at shops without formal diagnostic testing, which can result in mistreating diseases[35], and private medical sources lack formal regulation. The proportion of households in the private, mixed, and government health care categories was 27.30% (953/3491), 34.20% (1194/3491), and 38.50% (1344/2391), respectively. Similarly, the proportion of individuals in each category was 23.95% (3918/16357), 36.17% (5916/16357), and 39.88% (6523/16357), respectively.

6. Village intestinal helminth prevalence. An index of intestinal helminth prevalence was constructed to not only capture NTD coinfections in the study area, but also to represent varying environmental conditions and infection risk exposure. Our study area is endemic for intestinal schistosomiasis and hookworm [5]. These infections are geographically focused and have distinct transmission routes; schistosomiasis depends on contact with freshwater sources containing competent intermediate snail hosts and hookworm depends on contact with contaminated soil in the appropriate microclimatic conditions. Consequently, the village prevalence of each infection captures distinct village ecology and infrastructure [5]. Village prevalence was calculated as the percentage of all individuals sampled with at least one egg per gram of stool. A stratified random sample of ~60 people per village was used. Prevalence was measured before MDA and the study villages had not received MDA for 1.5 years prior to the sampling. Detailed methods for the participant sampling, parasitology, and measurement of village ecology and infrastructure are described in Chami *et al* [5]. We classified villages by prevalence of *S. mansoni* and hookworm infections, utilizing WHO categories of prevalence for MDA treatment [18]. High, moderate, and low *S. mansoni* village prevalence was >50%, ≤50% and ≥10%, and <10%, respectively. For hookworm, high, moderate, and low prevalence was >50%, ≤50% and ≥20%, and <20%, respectively. It should be noted that for low prevalence of hookworm, the WHO does not recommend MDA [18]. All individuals within a village were assigned the village prevalence category. There were five unique categories of prevalence for the 16,357 people in the 17 study villages. The number of villages and individuals per category are as follows: 1) high *S. mansoni* and high hookworm (1 village, 996 people); 2) high *S. mansoni*

and moderate hookworm (3 villages, 3431 people); 3) high *S. mansoni* and low hookworm (4 villages, 3842 people); 4) moderate *S. mansoni* and high hookworm (7 villages, 6235 people); and 5) moderate *S. mansoni* and moderate hookworm (2 villages, 1853 people). A summary of village characteristics by prevalence category is provided in Table S4.

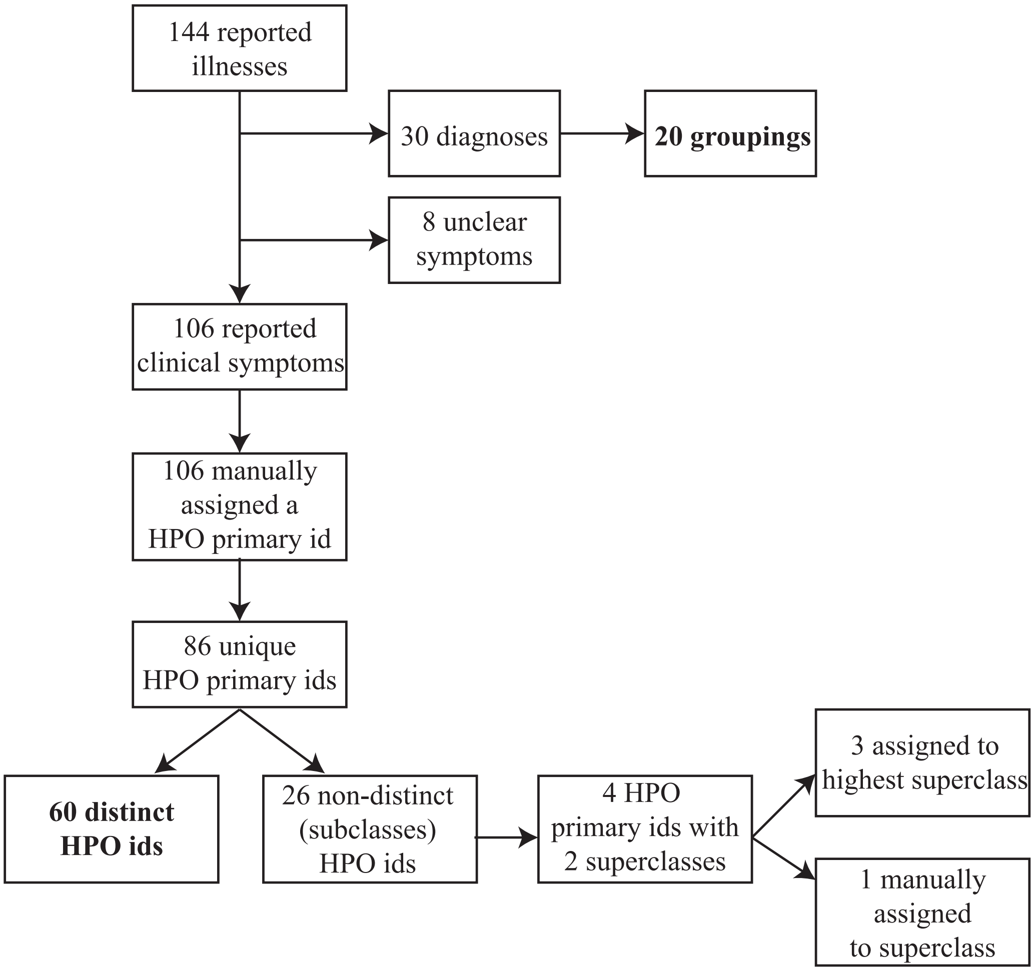

Figure S1: Mapping of reported symptoms/diseases to the human phenotype ontology (HPO).

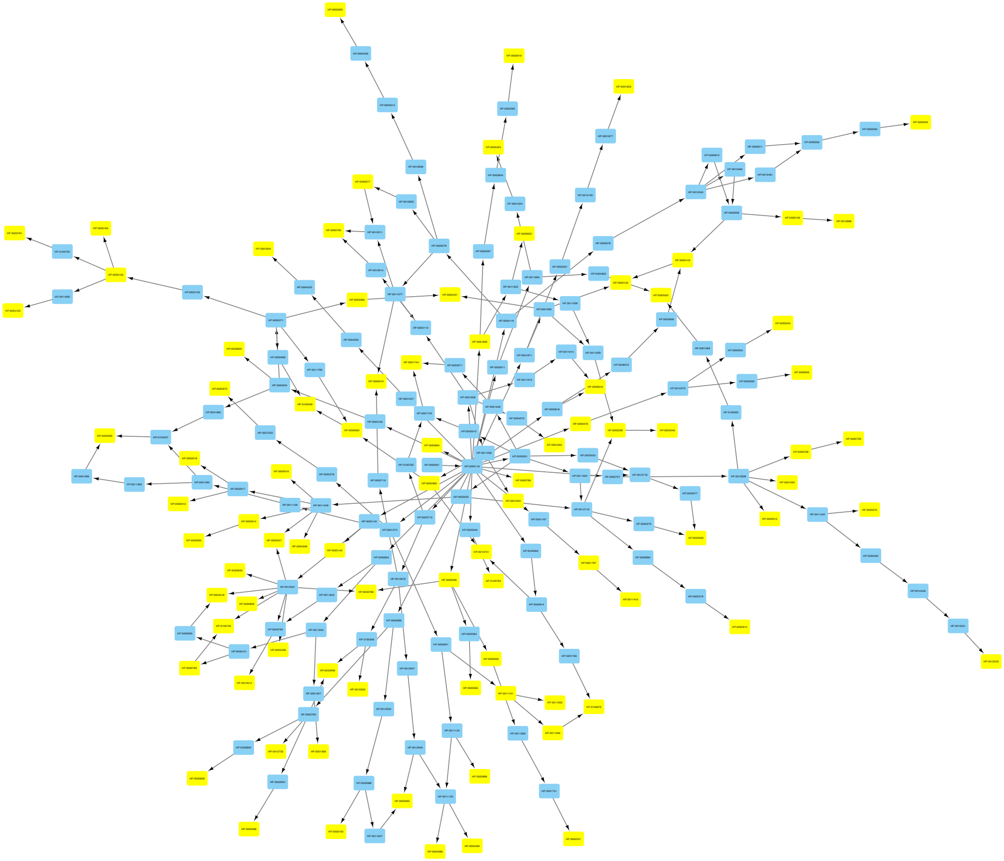

Figure S2: Directed acyclic graph, force-directed layout, of HPO for reported symptoms.

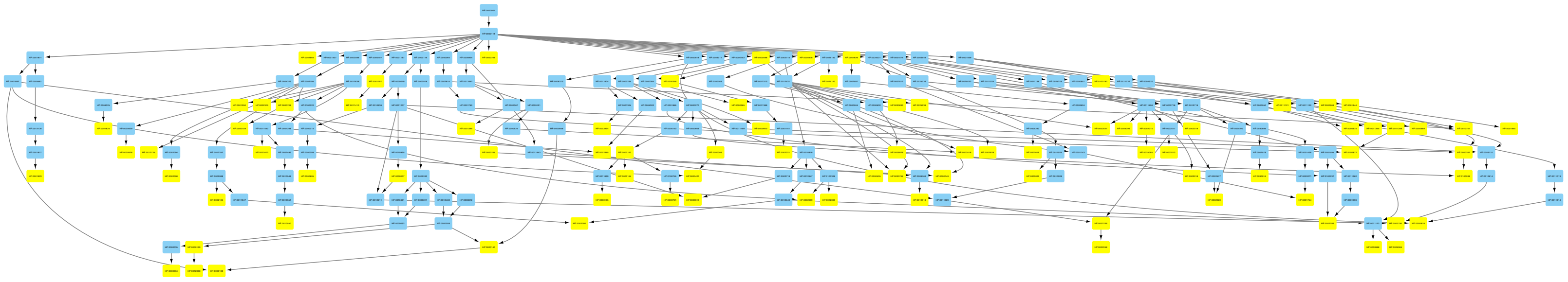

Figure S3: Directed acyclic graph, hierarchical layout, of HPO for reported symptoms.

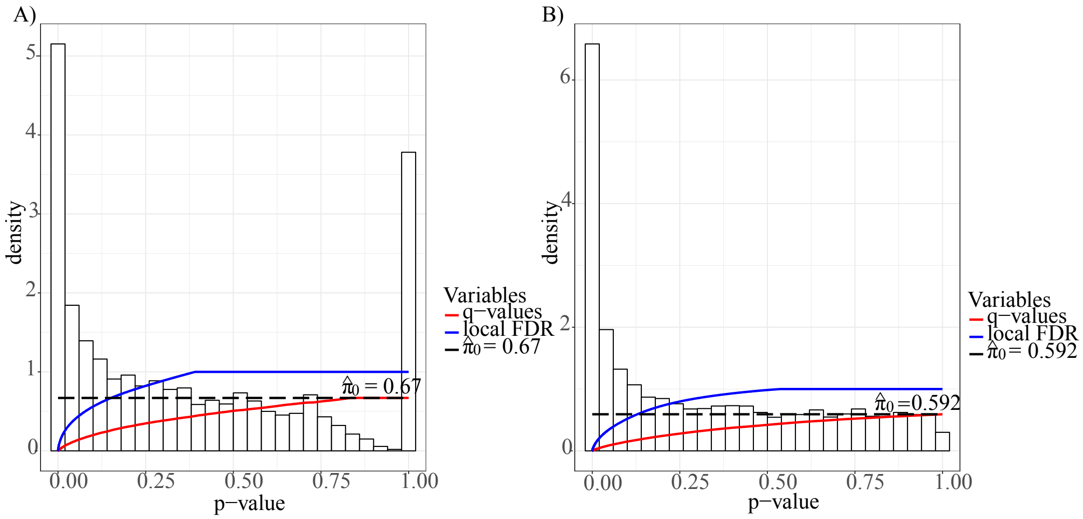

Figure S4: P-value distributions and estimated proportion of true null hypotheses for q-values. The q-values, local false discovery rate (FDR), and estimated proportion of true null hypotheses $\hat{\pi}_0$ were estimated for the RR and Spearman ρ correlations, see Comorbidity Network Methods for a detailed description. **A)** Histogram of p-values for RR. RR p-values display a U-shaped distribution because a two-sided significance test was run, though RR can only be ≥ 0. To account for the U-shaped distribution, the bootstrap method was used to estimate $\hat{\pi}_0$ as described in [36]. **B)** Histogram of p-values for Spearman ρ. The smoother method was used to estimate $\hat{\pi}_0$ as described in [14].

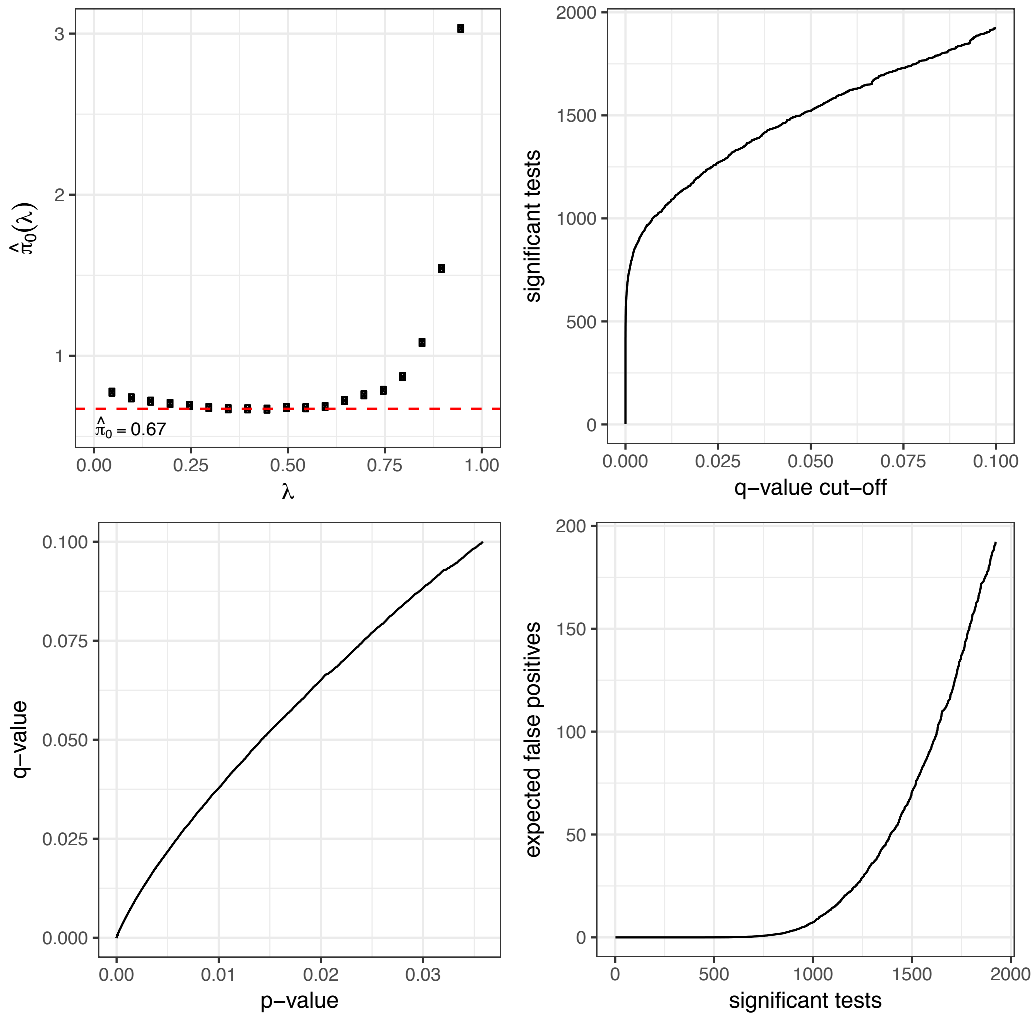

Figure S5: Descriptive statistics for relative risk q-values. $\hat{\pi}_0$ is the estimated proportion of true null hypotheses. λ is the tuning parameter.

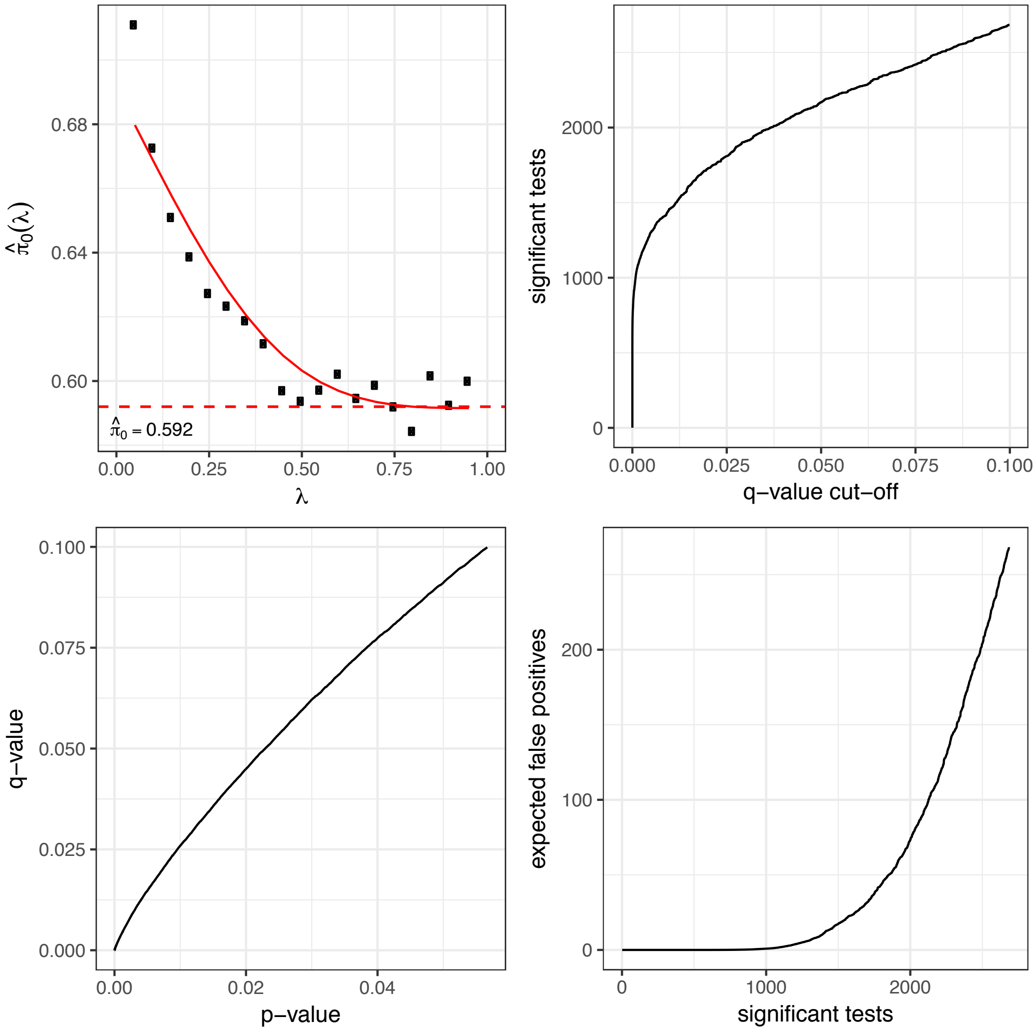

Figure S6: Descriptive statistics for Spearman ρ q-values. $\hat{\pi}_0$ is the estimated proportion of true null hypotheses. λ is the tuning parameter.

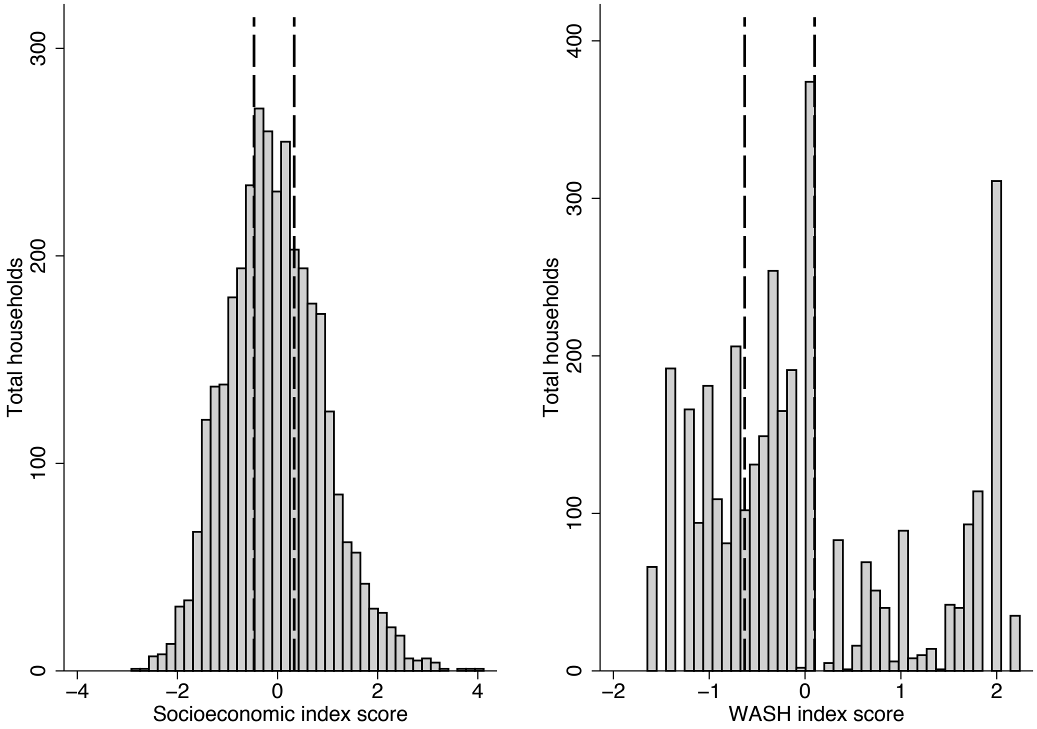

Figure S7: Principal component analysis scores from first component. Dashed lines represent cutoffs for the 3-level socioeconomic status and water, sanitation, and hygiene (WASH) indices.

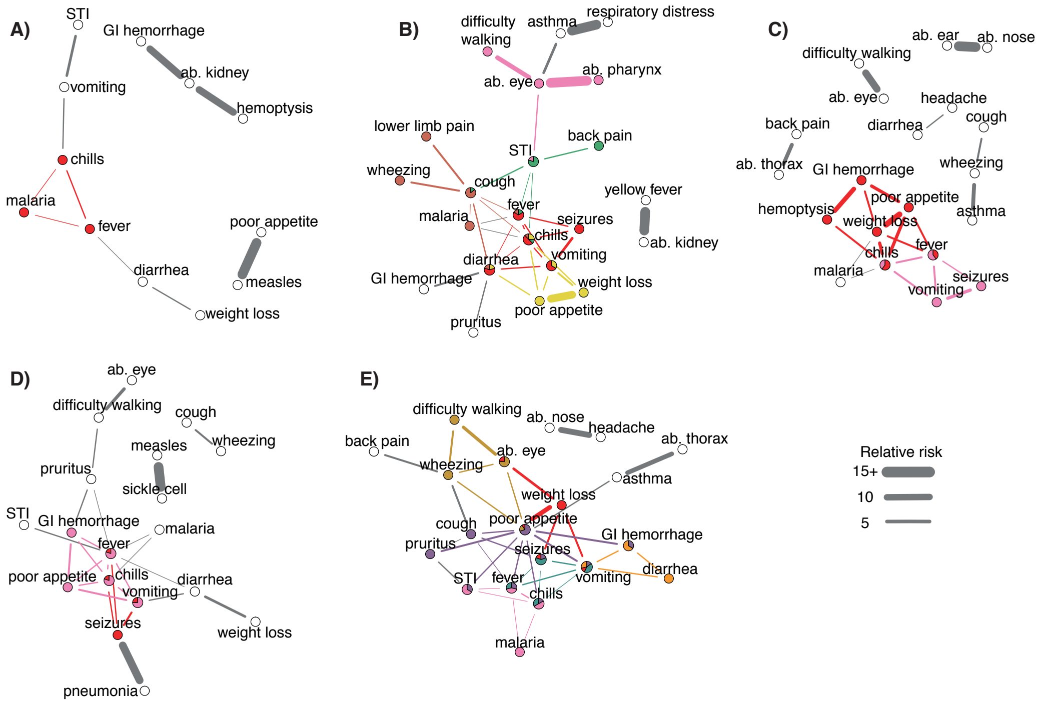

Figure S8: Comorbidity networks by village ecology. A) High *S. mansoni* prevalence, High hookworm prevalence. **B)** High *S. mansoni* prevalence, Moderate hookworm prevalence. **C)** High *S. mansoni* prevalence, Low hookworm prevalence. **D)** Moderate *S. mansoni* prevalence, Moderate hookworm prevalence. **E)** Moderate *S. mansoni* prevalence, High hookworm prevalence. Nodes represent reported symptoms/diseases; HPO terms are shown for symptoms. Abbreviations: ab. "abnormality of the", GI "astrointestinal", and STI "sexually transmitted infection". Edges shown had relative risk (RR) >1, $\phi > 0$, and q-value<0.05. Widths of edges are capped at a RR of 15. Colors correspond to link clusters.

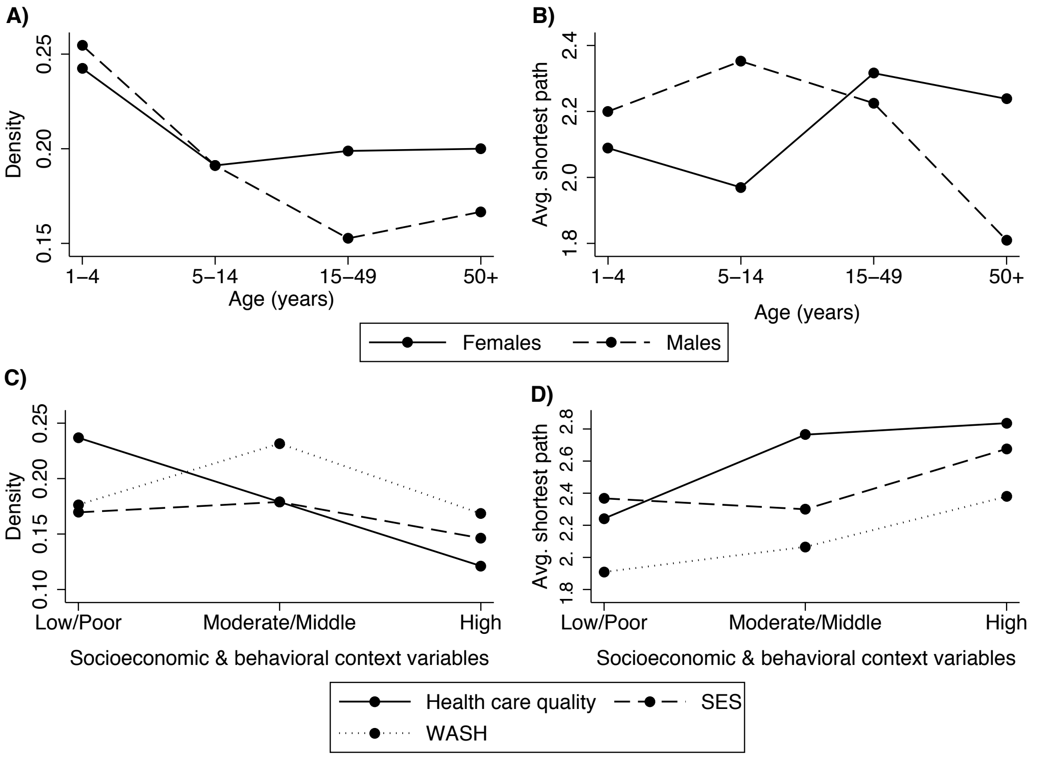

Figure S9: Trends in global network density and avg. shortest path. Density **A)** and average shortest path **B)** against age and gender comorbidity networks. Density **C)** and average shortest path **D)** against socioeconomic and behavioral contexts. Health care quality: low (private clinics only), middle (mixed care), and high (government health centres only). SES: socioeconomic status index. WASH: water, sanitation, and hygiene index.

Table S1: Descriptive statistics of ecological sub-group of comorbidity network analyses

Division	Total people reporting at least 1 illness	Reported symptoms/ diseases	Maximum possible comorbidities	Actual comorbidities (cooccurrence≥1)	Significant symptoms/ diseases (nodes)	Significant comorbidities (edges)
Village intestinal helminth prevalence						
High SM, High HW	802	45	990	180	12	10
High SM, Moderate HW	3051	64	2016	398	22	35
High SM, Low HW	3292	55	1485	278	20	22
Moderate SM, High HW	5511	72	2556	443	20	38
Moderate SM, Moderate HW	1746	54	1431	254	18	25

Table S2: Socioeconomic variable weights from first principal component

Variable	Value for dummies	Scoring coefficients
Council	0	-0.093
	1	0.723
Other status	0	-0.032
	1	0.706
Majority tribe	0	-0.078
	1	0.138
Muslim	0	0.007
	1	-0.015
Fishing household	0	0.168
	1	-0.376
Farming household	0	-0.236
	1	0.278
Education	N/A	0.208
Home ownership	0	-0.653
	1	0.112
Home electricity	0	-0.032
	1	0.381
Home quality score	N/A	0.241
Years in village	N/A	0.400

Table S3: Water, sanitation, and hygiene (WASH) variable weights from first principal component

Variable	Value for dummies	Scoring coefficients
Improved drinking water	0	-0.549
	1	0.248
Improved cooking water	0	-0.262
	1	0.669
Improved bathing water	0	-0.194
	1	0.797
Water availability/quantity	0	-0.137
	1	0.081
Water treatment	0	0.111
	1	-0.178
Soap availability	0	0.143
	1	-0.297
Improved sanitation	0	-0.028
	1	0.192

Table S4: Village characteristics by intestinal helminth prevalence

	Village intestinal helminth prevalence				
	High SM, High HW	High SM, Moderate HW	High SM, Low HW	Moderate SM, High HW	Moderate SM, Moderate HW
**Binary variables**	Frequency (Number of villages in each category)				
Village roads (3+)	1/1	1/3	1/4	4/7	1/2
Village centre >0.5 km from Lake Victoria	0/1	0/3	1/4	7/7	2/2
Rice paddy in village	1/1	2/3	2/4	6/7	2/2
Beach in village	1/1	3/3	1/4	1/7	1/2
Lake landing site (no beach) in village	0/1	0/3	1/4	5/7	0/2
**Continuous variables**	Average (std. dev.)				
Total households	194 (N/A)	279.67 (185.24)	208.50 (52.03)	190.71 (70.73)	188 (11.31)
Mean haversine distance (metres) between two households in the village	440.99 (N/A)	163.77 (31.03)	460.99 (106.13)	434.04 (77.95)	493.68 (205.04)

SM: *S. mansoni.* HW: hookworm. For prevalence classifications, see "Village intestinal helminth prevalence" section.

References

1. Hartung C, Lerer A, Anokwa Y, Tseng C, Brunette W, Borriello G. Open data kit: tools to build information services for developing regions. Proceedings of the 4th ACM/IEEE International Conference on Information and Communication Technologies and Development. London, United Kingdom: ACM; 2010. p. 1–12.
2. Köhler S, Vasilevsky NA, Engelstad M, *et al.* The Human Phenotype Ontology in 2017. *Nucleic Acids Res.* 2017; 45: D865–D76.
3. Cline MS, Smoot M, Cerami E, *et al.* Integration of biological networks and gene expression data using Cytoscape. *Nat. Protoc.* 2007; 2: 2366–82.
4. Schult DA, Swart P. Exploring network structure, dynamics, and function using NetworkX. Proceedings of the 7th Python in Science Conferences (SciPy); 2008. p. 11–6.
5. Chami GF, Fenwick A, Bulte E, *et al.* Influence of *Schistosoma mansoni* and Hookworm Infection Intensities on Anaemia in Ugandan Villages. *PLoS Negl. Trop. Dis.* 2015; 9: e0004193.
6. Hidalgo CA, Blumm N, Barabási A-L, Christakis NA. A dynamic network approach for the study of human phenotypes. *PLoS Comput. Biol.* 2009; 5: e1000353.
7. Fisher RA. The Logic of Inductive Inference. *J. Royal Stat. Soc*. 1935; 98: 39–82.
8. Gomez-Cabrero D, Menche J, Vargas C, *et al.* From comorbidities of chronic obstructive pulmonary disease to identification of shared molecular mechanisms by data integration. *BMC Bioinformatics* 2016; 17: 23.
9. Roque FS, Jensen PB, Schmock H, *et al.* Using electronic patient records to discover disease correlations and stratify patient cohorts. *PLoS Comput. Biol.* 2011; 7: e1002141.
10. Mehrotra DV, Chan ISF, Berger RL. A cautionary note on exact unconditional inference for a difference between two independent binomial proportions. *Biometrics* 2003; 59: 441–50.
11. Katz D, Baptista J, Azen SP, Pike MC. Obtaining confidence intervals for the risk ratio in cohort studies. *Biometrics* 1978; 34: 469–74.
12. Altman DG, Bland JM. How to obtain the P value from a confidence interval. *BMJ* 2011; 343: d2304.
13. Glickman ME, Rao SR, Schultz MR. False discovery rate control is a recommended alternative to Bonferroni-type adjustments in health studies. *J. Clin. Epidemiol.* 2014; 67: 850–7.
14. Storey JD, Tibshirani R. Statistical significance for genomewide studies. *Proc. Natl. Acad. Sci. U.S.A*. 2003; 100: 9440–5.
15. Storey JD. A direct approach to false discovery rates. *J. R. Stat. Soc. Series B. Stat. Methodol.* 2002; 64: 479–98.
16. Benjamini Y, Hochberg Y. Controlling the false discovery rate: A practical and powerful approach to multiple testing. *J. R. Stat. Soc. Series B. Stat. Methodol.* 1995; 57: 289–300.
17. Singer M, Bulled N, Ostrach B, Mendenhall E. Syndemics and the biosocial conception of health. *Lancet* 2017; 389: 941–50.
18. World Health Organization. Preventive Chemotherapy in Human Helminthiases, Coordinated Use of Anthelminthic Drugs in Control Interventions: A manual for Health Professionals and Programme Managers, 2006.
19. Lammie PJ, Fenwick A, Utzinger J. A blueprint for success: integration of neglected tropical disease control programmes. *Trends Parasitol.* 2006; 22: 313–21.

20. Chmiel A, Klimek P, Thurner S. Spreading of diseases through comorbidity networks across life and gender. *New J. Phys.* 2014; 16: 115013.
21. Wang H, Naghavi M, Allen C, *et al.* Global, regional, and national life expectancy, all-cause mortality, and cause-specific mortality for 249 causes of death, 1980-2015: a systematic analysis for the Global Burden of Disease Study 2015. *Lancet* 2016; 388: 1459–544.
22. Kabatereine NB, Vennervald BJ, Ouma JH, *et al.* Adult resistance to schistosomiasis mansoni: age-dependence of reinfection remains constant in communities with diverse exposure patterns. *Parasitology* 1999; 118: 101–5.
23. Ronsmans C, Graham WJ. Maternal mortality: who, when, where, and why. *Lancet*; 368: 1189–200.
24. Marmot M. Social determinants of health inequalities. *Lancet*; 365: 1099–104.
25. Chami GF, Kontoleon AA, Bulte E, *et al.* Profiling nonrecipients of mass drug administration for schistosomiasis and hookworm infections: a comprehensive analysis of praziquantel and albendazole coverage in community-directed treatment in Uganda. *Clin. Infect. Dis.* 2016; 62: 200–7.
26. Filmer D, Pritchett LH. Estimating wealth effects without expenditure data—or tears: an application to educational enrollments in states of India. *Demography* 2001; 38: 115–32.
27. Vyas S, Kumaranayake L. Constructing socio-economic status indices: how to use principal components analysis. *Health Policy Plan.* 2006; 21: 459–68.
28. Kolenikov S, Angeles G. Socioeconomic status measurement with discrete proxy variables: Is principal component analysis a reliable answer? *Rev. Income Wealth* 2009; 55: 128–65.
29. Schellenberg JA, Victora CG, Mushi A, *et al.* Inequities among the very poor: health care for children in rural southern Tanzania. *Lancet* 2003; 361: 561–6.
30. UNICEF. Strategy for Water, Sanitation, and Hygiene 2016-2030. New York: United Nations, 2016.
31. United Nations. Transforming our world: The 2030 agenda for sustainable development. A/RES/70/1. New York, New York: United Nations, 2015.
32. Strunz EC, Addiss DG, Stocks ME, Ogden S, Utzinger J, Freeman MC. Water, Sanitation, Hygiene, and Soil-Transmitted Helminth Infection: A Systematic Review and Meta-Analysis. *PLOS Medicine* 2014; 11: e1001620.
33. Rutebemberwa E, Pariyo G, Peterson S, Tomson G, Kallander K. Utilization of public or private health care providers by febrile children after user fee removal in Uganda. *Malar. J.* 2009; 8: 45.
34. Masaku J, Mwende F, Odhiambo G, *et al.* Knowledge, practices and perceptions of geo-helminthes infection among parents of pre-school age children of Coastal region, Kenya. *PLoS Negl. Trop. Dis.* 2017; 11: e0005514.
35. Källander K, Hildenwall H, Waiswa P, Galiwango E, Peterson S, Pariyo G. Delayed care seeking for fatal pneumonia in children aged under five years in Uganda: a case-series study. *Bull. World Health Organ.* 2008; 86: 332–8.
36. Storey JD, Taylor JE, Siegmund D. Strong control, conservative point estimation and simultaneous conservative consistency of false discovery rates: a unified approach. *J. R. Stat. Soc. Series B. Stat. Methodol.* 2004; 66: 187–205.
